# An uncertainty-aware ergonomic risk assessment framework using Interval-Valued Fermatean Fuzzy Sets and hybrid MCDM

**DOI:** 10.1038/s41598-026-46701-9

**Published:** 2026-04-04

**Authors:** Ayça Zenginoğlu, Yusuf Kuvvetli

**Affiliations:** 1 Technology Transfer Office Coordinatorship, Aksaray University, Aksaray, 68100 Turkey; 2 Department of Industrial Engineering, Engineering Faculty, Cukurova University, Adana, 01330 Turkey

**Keywords:** Ergonomic risk assessment, Musculoskeletal disorders, Interval-Valued Fermatean Fuzzy Sets, Multi-criteria decision-making, Manufacturing systems, Engineering, Health care, Mathematics and computing

## Abstract

Musculoskeletal disorders (MSDs) pose a significant occupational health concern in manufacturing, where traditional ergonomic risk assessment methods (RULA, REBA, OWAS, OCRA, QEC, and SI) are limited by subjectivity and the inadequate handling of interdependent criteria. Existing research reveals a notable gap in integrated frameworks that systematically combine multiple assessment methods under uncertainty. This study introduces a novel approach that integrates Interval-Valued Fermatean Fuzzy Sets (IVFFS) with multi-criteria decision-making (MCDM) techniques for systematic ergonomic evaluation. The framework incorporates IVFFS–PIPRECIA for uncertainty-aware factor weighting, IVFFS–MAIRCA for comparative method ranking, and an ENTROPY formulation that treats tasks as criteria to enhance objectivity. The results show that the force requirement and automation compatibility are the most influential ergonomic factors, whereas REBA, OCRA, and OWAS demonstrate superior overall performance. Comparative findings further reveal substantial variation across single-method evaluations: posture-centric tools disproportionately emphasize certain risks, whereas the integrated model uncovers overlooked high-risk activities by aggregating the complementary strengths of different methods. The proposed framework advances ergonomic risk management by integrating advanced fuzzy logic and MCDM techniques, providing a transparent, systematic decision-support tool for practitioners and industrial stakeholders. It supports evidence-based workplace design and contributes to safer, more efficient manufacturing systems.

**Supplementary Information:**

The online version contains supplementary material available at 10.1038/s41598-026-46701-9.

## Introduction and literature background

### Problem context and motivation

Work-related musculoskeletal disorders (WMSDs) remain among the most prevalent occupational health problems in manufacturing and industrial environments, leading to productivity losses, increased absenteeism, and long-term health impairments^1,2^. To mitigate these risks, ergonomic risk assessment has become an essential component of occupational safety management, particularly in production systems characterized by manual handling, repetitive tasks, and physically demanding operations^3,4^.

Widely used ergonomic assessment tools—such as REBA, RULA, OWAS, OCRA, QEC, and SI—provide structured frameworks for identifying postural loads, repetitive movements, and biomechanical stressors^5–9^. Their practical applicability and ease of use have facilitated their widespread adoption in industrial practice. However, these methods rely heavily on expert judgment and discrete scoring schemes, which often fail to capture the uncertainty, hesitation, and variability inherent in real-world ergonomic evaluations^10,11^.

In complex production environments, ergonomic risk is rarely driven by a single factor. Instead, it emerges from the interaction of multiple criteria, including posture, force exertion, repetition frequency, environmental conditions, and task-specific constraints. The subjective nature of expert assessments, combined with incomplete or ambiguous information, further complicates the reliable quantification of risk^9,12–14^. These challenges underscore the need for decision-support frameworks that systematically integrate multiple ergonomic criteria while explicitly accounting for uncertainty in expert judgments.

### Limitations of existing ergonomic assessment approaches

Although traditional ergonomic assessment methods have proven valuable for preliminary risk screening, several limitations restrict their effectiveness in complex industrial decision-making contexts. First, most methods evaluate ergonomic risk factors independently, offering limited ability to model interdependencies among criteria or to prioritize competing ergonomic concerns within a unified framework^15–17^.

Second, comparisons among different ergonomic assessment methods are typically conducted in isolation, without systematic consideration of how varying methodological assumptions influence overall risk rankings. As a result, practitioners often face inconsistent or conflicting conclusions when multiple methods are applied to the same operational setting^18–20^.

Third, uncertainty and expert hesitation are usually treated implicitly or ignored altogether. Conventional approaches rely on crisp scores or narrowly defined linguistic categories, which inadequately represent the confidence ranges and ambiguity inherent in expert-based evaluations^21–23^. This limitation becomes particularly critical when assessments involve complex tasks, heterogeneous working conditions, or limited observational data.

To address these challenges, MCDM techniques have increasingly been integrated into ergonomic risk assessment. MCDM frameworks enable simultaneous consideration of multiple criteria and provide structured mechanisms for weighting and ranking alternatives^10,23,24^. However, the effectiveness of such frameworks depends on how uncertainty and expert hesitation are modeled within the decision-making process.

Recent research has also explored AI-driven and digital ergonomic assessment systems. For instance, Menanno et al.^25^ proposed a 3D pose estimation system integrated with collaborative robotics, while Chatzis et al.^26^ developed a deep learning-based framework for automatic ergonomic risk detection. Similarly, Alenjareghi et al.^27^ reviewed wearable sensor technologies combined with machine learning techniques for WMSD prevention in Industry 4.0 environments, and Mirzahosseininejad et al.^28^ introduced a deep learning architecture for real-time ergonomic risk prediction in human–robot collaboration contexts.

Complementing these developments, recent studies have also investigated ergonomic risk assessment in applied industrial and occupational contexts. For example, Sakinala et al.^29^ employed fuzzy modeling within participatory ergonomics to enhance operator safety in mining environments. Additionally, ergonomic evaluations have been conducted in assistive technologies such as exoskeleton systems Sado et al.^30^ and in occupational health studies examining the relationships between ergonomic risk factors and musculoskeletal disorders Mohammadian et al.^31^. These developments further highlight the growing importance of systematic and data-driven ergonomic assessment approaches across diverse application domains.

Although these studies represent significant advancements in real-time monitoring, automated posture analysis, and predictive ergonomics, their primary focus remains on dynamic risk detection and classification. In contrast, comparatively limited attention has been devoted to structured, uncertainty-aware prioritization of ergonomic assessment methods at the decision-support level. The present study addresses this methodological gap by focusing on method selection and comparative evaluation under epistemic uncertainty rather than real-time risk prediction.

### Fuzzy set extensions in ergonomic decision-making

Fuzzy set theory has been widely adopted to enhance ergonomic risk assessment by enabling the representation of linguistic uncertainty and subjective judgments^17,32^. Classical fuzzy sets introduce membership functions to model vague concepts such as “low” or “high” risk, improving flexibility over crisp scoring schemes. Nevertheless, their single-membership structure limits the explicit representation of expert hesitation^21,33^.

To overcome this limitation, intuitionistic fuzzy sets (IFSs) were proposed by incorporating both membership and non-membership degrees, along with a hesitation component^21^. This extension allows experts to express partial uncertainty, which is particularly relevant in ergonomic assessments involving ambiguous or incomplete information. Pythagorean fuzzy sets (PFSs) further expanded the feasible decision space by relaxing the membership constraints, enabling more flexible modeling of conflicting expert opinions^18^.

More recently, Fermatean fuzzy sets (FFS) have been introduced to provide an even larger representational space through a cubic constraint on membership and non-membership degrees^22,34^. This formulation enhances the ability to capture deep uncertainty and extreme hesitation, which are frequently encountered in complex decision-making environments. Building on this framework, Interval-Valued Fermatean Fuzzy Sets (IVFFS) allow both membership and non-membership degrees to be expressed as intervals, thereby modeling confidence ranges and variability in expert judgments^24,35,36^. Compared to classical, intuitionistic, and Pythagorean fuzzy representations, IVFFS offer a larger feasible decision space and enable a more flexible representation of expert hesitation through interval-based membership structures. This is particularly relevant in ergonomic risk assessment, where expert judgments are often imprecise and context-dependent. Therefore, IVFFS is adopted in this study as a suitable framework for capturing uncertainty and variability in expert evaluations.

In MCDM applications, IVFFS has been successfully applied in domains such as energy planning, sustainable manufacturing, healthcare decision-making, and equipment selection, where uncertainty and expert hesitation play critical roles^37–40^. Despite these advances, the application of IVFFS within ergonomic risk assessment remains limited, and its potential to enhance objectivity and robustness in this domain has not been systematically explored^18,19^. Previous studies have applied various ergonomic risk assessment methods across different sectors, often combined with fuzzy and MCDM-based solution techniques. A structured overview of existing fuzzy and MCDM-based ergonomic risk assessment studies, including the ergonomic factors considered, application domains, and solution methods, is provided in Supplementary Table S1, based on a comprehensive review of the relevant literature, which includes additional relevant references^41–44^. 

As summarized in Supplementary Table S1, most recent studies have focused on specific ergonomic risk dimensions or single assessment tools. In contrast, comprehensive comparative evaluations of multiple ergonomic assessment methods under uncertainty remain limited, particularly in machining environments.

### Research gaps and contributions

Despite the growing body of research on ergonomic risk assessment and fuzzy multi-criteria decision-making (MCDM) approaches, several critical gaps remain insufficiently addressed. First, existing ergonomic studies predominantly rely on conventional fuzzy extensions or crisp evaluations, which inadequately capture expert hesitation and epistemic uncertainty inherent in real-world industrial assessments. Second, traditional ergonomic assessment methods are generally applied in isolation, without a unified analytical framework that enables systematic, context-sensitive comparison among alternative methods. Third, although advanced fuzzy representations have been developed in other decision-making domains, their methodological implications for ergonomic method selection and uncertainty-aware decision structuring have not been comprehensively examined.

While recent research emphasizes AI-driven real-time ergonomic monitoring and predictive risk detection, comparatively limited attention has been devoted to uncertainty-aware decision-support frameworks that assist practitioners in systematically selecting among alternative ergonomic assessment methods. The proposed study addresses this methodological gap by focusing on structured method prioritization rather than automated risk prediction.

Given these gaps, the present study reconceptualizes ergonomic assessment as a structured decision architecture that integrates uncertainty-aware criterion weighting, comparative-method prioritization, and operational risk aggregation. In response to these gaps, the present study proposes an uncertainty-aware decision-support framework designed to systematically prioritize ergonomic risk factors, support the selection of context-appropriate assessment methods, and enable structured operational risk evaluation in industrial environments. The study is guided by the following research questions:

#### RQ1

Which ergonomic risk factors emerge as the most influential when evaluated under uncertainty?

#### RQ2

How do traditional ergonomic risk assessment methods compare when systematically analyzed within a unified fuzzy MCDM framework?

#### RQ3

How does the enhanced representational capacity of an IVFFS improve uncertainty modeling in ergonomic risk assessment compared with conventional fuzzy approaches?

To address these questions, this study makes several theoretical and practical contributions. From a theoretical perspective, the study extends fuzzy MCDM applications in ergonomics from conventional risk-factor ranking toward structured method selection under uncertainty in expert evaluations. It introduces an integrated IVFF-based PIPRECIA–MAIRCA architecture for uncertainty-aware criterion weighting and method prioritization, combined with an ENTROPY-based operational aggregation stage. This structure preserves hesitation information throughout the decision process and conceptualizes ergonomic assessment as a layered analytical system rather than a single-stage evaluation.

From a practical perspective, the proposed framework provides decision-makers with a transparent and implementable mechanism for prioritizing ergonomic assessment methods before their operational application. By reducing reliance on subjective familiarity-based selection and enabling spreadsheet-compatible implementation, the framework enhances methodological rigor while remaining feasible for real-world industrial contexts.

Based on the identified research gaps and formulated research questions, the following section presents the methodological structure of the proposed IVFF–MCDM framework. The remainder of the paper is organized as follows: Sect. ''Materials and methods'' details the methodological framework; Sect. ''Case study and numerical results'' describes the industrial case study; Sect.  "Comparative and robustness analysis" reports the empirical results; Sect. "Discussion" discusses the findings in relation to the research questions; and Sect. "Limitations and future research directions" concludes the study and outlines future research directions.

## Materials and methods

### Problem description

The increasingly competitive business climate pressures manufacturing companies to deliver efficient, sustainable production processes. However, poor ergonomic working conditions continue to pose a challenge, leading to lower employee productivity, higher defect and scrap rates, and higher operational costs. These indirect impacts can negatively affect a company’s profitability and erode its competitive position^1,2^.

Effectively managing ergonomic risk is therefore crucial. Once these risks exceed acceptable levels, workers’ health and performance are compromised by physical strain and fatigue. However, existing ergonomic assessment approaches often rely on single methods or subjective judgments, which may fail to capture the multidimensional and uncertain nature of ergonomic risks in complex manufacturing processes. In response, this study proposes a systematic, integrated evaluation framework that combines multiple assessment techniques to prevent ergonomic risk factors from reaching critical levels, thereby supporting more sustainable, efficient production processes and improving working conditions.

In the context of ergonomic risk assessment, uncertainty stems primarily from subjective expert judgments, linguistic ambiguity in exposure evaluation, and incomplete knowledge regarding the cumulative and context-dependent nature of human-factor-related risks. This uncertainty does not represent stochastic variability; rather, it reflects epistemic imprecision and evaluative hesitation inherent in expert-based assessments. The use of IVFFS within the proposed framework enables explicit modeling of this epistemic and linguistic uncertainty, preserving hesitation while maintaining analytical consistency.

### The proposed framework

**Fig. 1:**
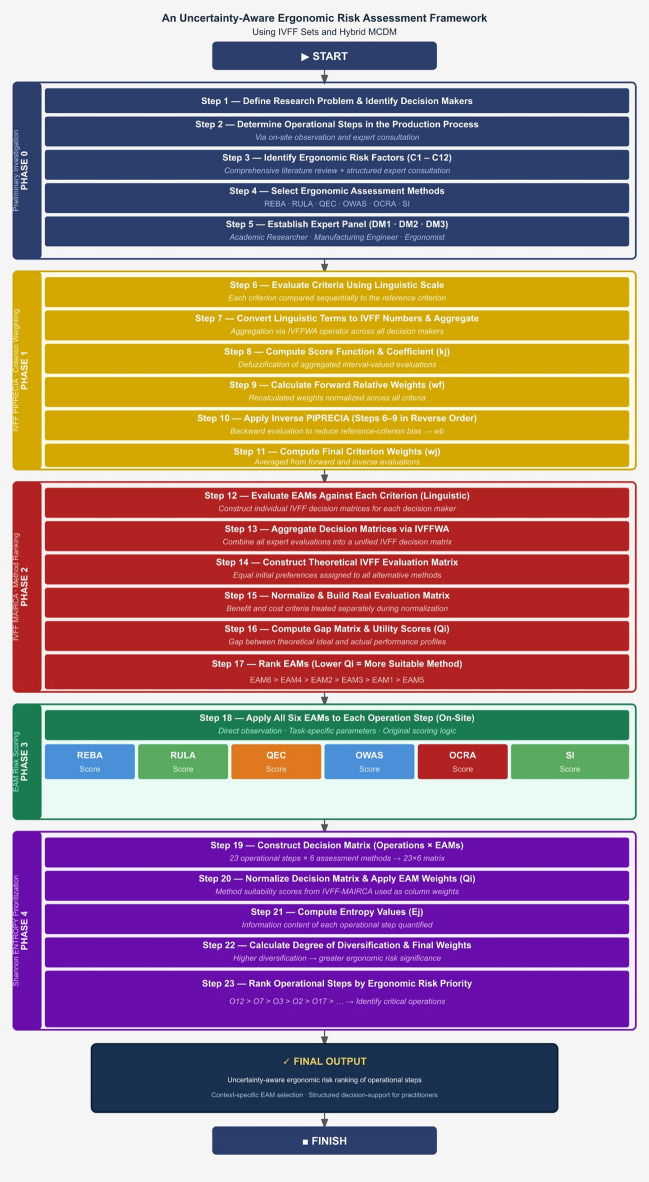
Framework of the proposed approach. This figure presents the integrated ergonomic risk assessment framework, illustrating the sequential analytical structure from the preliminary investigation and identification of ergonomic risk factors to method weighting, ergonomic risk scoring, and final prioritization of operational risks.

The proposed framework operationalizes the uncertainty-aware perspective described above by structurally embedding expert-derived epistemic uncertainty into the weighting and evaluation stages. As illustrated in Fig. 1, the framework is organized as a layered decision architecture composed of interrelated analytical stages that function as a coherent evaluation system.

Initially, the operational steps of the production process and the relevant ergonomic risk factors are identified through expert consultations and a comprehensive literature review. In the first analytical stage, commonly used ergonomic assessment methods (REBA, RULA, OCRA, QEC, SI, and OWAS) are applied to these operational steps to generate quantitative ergonomic risk scores, forming the foundational data layer of the analysis.

In the second stage, IVFF–PIPRECIA is employed to determine uncertainty-aware weights for the identified ergonomic risk factors based on expert judgments. These weights are then incorporated into the IVFF–MAIRCA procedure to evaluate and prioritize the relative suitability of the ergonomic assessment methods within a structured comparative framework.

In the final stage, the Shannon ENTROPY method integrates the method suitability outcomes with the generated ergonomic risk scores to derive an objective prioritization of production operations and identify the most critical ergonomic risk areas.

Thus, each methodological component fulfills a distinct and complementary analytical function within the unified decision-support structure, contributing to an integrated and coherent evaluation architecture. A schematic representation of the proposed framework is provided in Fig. 1.

To enhance interpretability, the overall workflow of the proposed framework can be summarized as follows:

(i) Generation of operation-level ergonomic risk scores using ergonomic risk assessment methods,
(ii) Uncertainty-aware weighting of ergonomic criteria via IVFF–PIPRECIA,
(iii) Comparative prioritization of ergonomic assessment methods using IVFF–MAIRCA, and.
(iv) Objective integration and operational risk prioritization through the ENTROPY method.

This layered structure ensures functional coherence while maintaining methodological transparency.

### Phase 1: preliminary investigation and preparation process

In the preliminary phase, an expert panel was established to ensure reliable and balanced evaluations throughout the study. The panel consisted of three decision makers (DMs) with complementary expertise: DM1, an academic with more than ten years of experience in research and higher education; DM2, an engineer with eight years of hands-on industrial experience in machining and manufacturing operations; and DM3, an ergonomist specializing in occupational safety and risk management with seven years of professional experience. The selection of decision makers was based on their domain-specific expertise, professional experience, and demonstrated familiarity with ergonomic risk assessment methods. In addition, particular emphasis was placed on ensuring disciplinary complementarity, enabling the integration of academic, engineering, and ergonomics perspectives within the evaluation process. Their combined expertise covers widely used ergonomic risk assessment methods (e.g., REBA, RULA, OCRA, and QEC) and key ergonomic risk factors, including posture, force, repetition, and environmental conditions. The inclusion of experts from diverse professional backgrounds ensures that the evaluation is conducted from a multidisciplinary perspective, thereby enhancing the robustness and credibility of the analysis.

Following the establishment of the expert panel, the operational steps of the production process were identified through on-site observations and consultations with domain experts. The analyzed manufacturing process encompasses the main production stages, including raw material inspection, machining and forming operations, surface treatments, assembly, and final quality control. These operational steps were selected to represent tasks with varying physical demands and ergonomic exposures, forming the empirical basis for subsequent ergonomic risk analysis.

Building upon the identified operational context, relevant ergonomic risk factors were determined through an integrated approach combining a comprehensive literature review and expert consultation. The selected factors address multiple dimensions of ergonomic exposure, including physical load requirements, posture-related difficulties, repetitive movement patterns, and environmental conditions that may affect workers’ health and performance. Table 1 presents the complete set of ergonomic risk criteria with their operational definitions and supporting literature.

**Table 1:** Ergonomic risk criteria and definitions.

Code	Criteria	Reference	Description
C1	Force requirement	Koppiahraj et al.^13^; Khandan et al.^45^	It analyzes activities that require physical effort from the operator, such as lifting, pushing, or pulling.
C2	Transport and lifting analysis	Koppiahraj et al.^13^; Khandan et al.^45^	Evaluate the operator’s physical difficulties while handling and lifting heavy or bulky objects.
C3	Analyzing repetitive movements	Koppiahraj et al.^13^; Delice and Can^15^, ; Kose et al.^18^; Khandan et al.^45^	It analyzes the physical strain on the operator caused by repetitive movements.
C4	Posture difficulties	Koppiahraj et al.^13^; Khandan et al.^45^	It identifies ergonomic risks by evaluating the operator’s body positions, such as bending and turning, in detail.
C5	Vibration exposure	Kulaç and Kiraz^19^; Khandan et al.^45^; Kamala et al.^46^;	It analyses the impact of mechanical vibrations on operator health and musculoskeletal strain
C6	Noise exposure	Kulaç and Kiraz^19^; Khandan et al.^45^; Kamala et al.^46^;	It evaluates the effect of workplace noise levels on operator performance and auditory health.
C7	Temperature exposure	Kulaç and Kiraz^19^; Khandan et al.^45^; Kamala et al.^46^;	It assesses the impact of thermal conditions (heat/cold) on operator comfort and productivity.
C8	Capacity for detail in body position analysis	David^9^; Roman-Liu^12^	It refers to the method’s ability to analyze the operator’s body positions in detail.
C9	Ease of use and practicality	Delice ve Can^15^; Kulaç and Kiraz^19^; David^9^; Roman-Liu^12^	It demonstrates the method’s rapid applicability and the ease with which its results can be interpreted.
C10	Equipment compatibility	David^9^; Roman-Liu^12^	Evaluates whether the method is compatible with existing equipment and working conditions.
C11	Precision in scoring risk levels	David^9^; Roman-Liu^12^	It refers to the method’s ability to accurately and precisely assess ergonomic risks.
C12	Automation and digitalization compatibility	Kulaç and Kiraz^19^,	It expresses the compatibility of the preferred method with automation and digitalization technologies.

To enable a comprehensive and comparable ergonomic assessment, six widely recognized ergonomic risk assessment methods—REBA, RULA, OCRA, QEC, SI, and OWAS—were selected for integration into the proposed framework. The selection was based on (i) their extensive validation and application in manufacturing environments, (ii) complementary focus areas addressing different risk dimensions, and (iii) recommendations from the expert panel considering the specific characteristics of the analyzed production process. Table 2 provides a comparative overview of these methods, highlighting their main characteristics, advantages, and limitations.

**Table 2:** Analyzed ergonomic risk assessment methods and definitions.

Method	Advantages	Disadvantages	Reference
REBA (EAM1)	- Quick and easy to apply. - Evaluate multiple body parts and repetitive activities. - Considers postural and lifting risks.	- No sub-scores for body parts. - Ignores environmental or psychosocial factors.	Hignett and McAtamney^6^
RULA (EAM2)	- Focused on upper extremities, neck, and trunk. - Effective for repetitive workload tasks.	- Limited to upper-body tasks. - Excludes environmental and psychosocial factors.	McAtamney and Corlett^5^
QEC (EAM3)	- Includes environmental and psychosocial risks. - Combines observer and worker feedback.	- Limited industrial use. - Lack of detailed scoring for some body parts.	Bidiawati and Suryani^47^
OWAS (EAM4)	- Effective for heavy industry postures and load handling. - Simple risk categorization.	- Ignores environmental and psychosocial factors. - Overlooks subtle posture differences.	Karhu et al.^8^
OCRA (EAM5)	- Comprehensive tasks - Includes environmental and psychosocial factors.	- Requires time-consuming studies. - Costly for large-scale assessments.	Occhipinti^7^
SI (EAM6)	- Targets upper extremity risks. - Provides quantitative scores.	- Focused only on upper extremities. - Not suitable for full-body assessments.	Moore and Garg^48^

Following the establishment of the expert panel and the identification of ergonomic risk factors, the next phase formalizes expert evaluations within an uncertainty-aware mathematical structure. This transition marks the shift from qualitative expert input to structured quantitative modeling.

### Phase 2: Prioritization of ergonomic risk factors with IVFF-PIPRECIA

In this phase, the ergonomic risk factors identified in Phase 1 are prioritized within an uncertainty-aware mathematical framework. Since expert evaluations inherently contain hesitation and linguistic ambiguity, an IVFF structure is adopted to formally model this uncertainty. The following subsection introduces the theoretical foundations of IVFFS before presenting the integrated IVFF–PIPRECIA weighting procedure.

#### Interval-valued fermatean fuzzy set (IVFFS)

The Fermatean fuzzy set (FFS) extends classical fuzzy set theory by providing a more flexible structure for modeling uncertainty and expert hesitation^49^. It jointly considers membership, non-membership, and hesitation degrees under a cubic constraint. The IVFFS builds on this by representing membership and non-membership degrees as intervals within the range [0, 1], thereby enhancing precision and enabling better modeling of uncertainty^23,35^. In line with the considerations outlined in Sect. "Fuzzy set extensions in ergonomic decision-making", IVFFS is adopted in this study to effectively capture uncertainty and hesitation in expert evaluations.

IVFFS has been shown to be particularly effective in applications such as information integration, multi-criteria decision-making, and risk analysis, where uncertainty and expert hesitation are prominent^23,33^. This representation enables more accurate modeling of expert preferences and uncertainty, leading to more reliable decision-making outcomes.

Equation (1) defines IVFF *A* as an extension of FFs, where Z denotes a non-empty finite set.

1 $$\:A=\:\left\{<\left[{\mu\:}_{A}^{L}\:\left(z\right),\:{\mu\:}_{A}^{U}\:\:\left(z\right)\:\right]\:\left[{\vartheta\:}_{A}^{L}\:\:\left(z\right),\:{\vartheta\:}_{A}^{U}\:\left(z\right)\:\right]\:>|\:z\in\:Z\:\right\}$$

where $\:0\:\le\:{\mu\:}_{A}^{L}\:\left(z\right)\:\le\:{\mu\:}_{A}^{U}\:\:\left(z\right)\le\:1,\:\:0\:\le\:{\vartheta\:}_{A}^{L}\:\:\:\left(z\right)\:\le\:{\vartheta\:}_{A}^{U}\:\:\left(z\right)\le\:1,$ $\:0\:\le\:({{\mu\:}_{A}^{U}\:\:\left(z\right))}^{3}+\:{{(\vartheta\:}_{A}^{U}\:\:\left(z\right))}^{3}\le\:1$. Additionally, the degree of hesitation of an element z with respect to the IVFF set A is defined as follows (Eq. 2).

2 $$\:{\pi\:}_{A}\left(z\right)=\left[{\pi\:}_{A}^{L}\left(z\right),\:{\pi\:}_{A}^{U}\left(z\right)\right]$$

where

3 $$\:{\pi\:}_{A}^{L}\left(z\right)=\sqrt[3]{1-{\left({\mu\:}_{A}^{U}\:\:\left(z\right)\right)}^{3}-{\left({\vartheta\:}_{A}^{U}\:\left(z\right)\right)}^{3}}$$

4 $$\:{\pi\:}_{A}^{U}\left(z\right)=\sqrt[3]{1-{\left({\mu\:}_{A}^{L}\:\:\left(z\right)\right)}^{3}-{\left({\vartheta\:}_{A}^{L}\:\left(z\right)\right)}^{3}}$$

Let an interval-valued fermatean fuzzy number (IVFFN) have the form as $\:f=\left(\left[{\mu\:}_{L},{\mu\:}_{U}\right],\left[{\vartheta\:}_{L},{\vartheta\:}_{U}\right]\right)$. For this IVFFN, the score function (*SF (f))* for defuzzification (crisp) is given by^23,32,33,46^.

$$\:SF\left(f\right)=\frac{1}{2}\left[{\left({\mu\:}^{L}\right)}^{3}+{\left({\mu\:}^{U}\right)}^{3}-{\left({\vartheta\:}^{L}\right)}^{3}-{\left({\vartheta\:}^{U}\right)}^{3}\right]\:\:\in\:\left[-\mathrm{1,1}\right].$$

Let $\:{f}_{1}=\left(\left[{\mu\:}_{1L},{\mu\:}_{1U}\right],\left[{\vartheta\:}_{1L},{\vartheta\:}_{1U}\right]\right)$ and $\:{f}_{2}=\left(\left[{\mu\:}_{2L},{\mu\:}_{2U}\right],\left[{\vartheta\:}_{2L},{\vartheta\:}_{2U}\right]\right)\:$and there are two IVFFNs, and *λ* is a positive real number. The arithmetic operations between these numbers are defined below.

- $$\:{f}_{1}\oplus\:{f}_{2}=\left[\sqrt[3]{{\mu\:}_{1L}^{3}+{\mu\:}_{2L}^{3}-{\mu\:}_{1L}^{3}{\mu\:}_{2L}^{3}}\:,\sqrt[3]{{\mu\:}_{1U}^{3}+{\mu\:}_{2U}^{3}-{\mu\:}_{1U}^{3}{\mu\:}_{2U}^{3}}\right],\left[{\vartheta\:}_{1L}{\vartheta\:}_{2L},{\vartheta\:}_{1U}{\vartheta\:}_{2U}\right]$$
- $$\:{f}_{1}⨂{f}_{2}=\left(\left[{\mu\:}_{1L}{\mu\:}_{2L},{\mu\:}_{1U}{\mu\:}_{2U}\right]\:,\left[\sqrt[3]{{\vartheta\:}_{1L}^{3}+{\vartheta\:}_{2L}^{3}-{\vartheta\:}_{1L}^{3}{\vartheta\:}_{2L}^{3}}\:,\:\sqrt[3]{{\vartheta\:}_{1U}^{3}+{\vartheta\:}_{2U}^{3}-{\vartheta\:}_{1U}^{3}{\vartheta\:}_{2U}^{3}}\right]\right)$$
- $$\:{{\uplambda\:}f}_{1}=\left(\left[\sqrt[3]{1-{\left(1-{\mu\:}_{1L}^{3}\right)}^{{\uplambda\:}}},\sqrt[3]{1-{\left(1-{\mu\:}_{1U}^{3}\right)}^{{\uplambda\:}}}\right],\left[{\vartheta\:}_{1L}^{{\uplambda\:}},{\vartheta\:}_{1U}^{{\uplambda\:}}\right]\right)$$
- $$\:{f}_{1}^{{\uplambda\:}}=\left(\left[{\mu\:}_{1L}^{{\uplambda\:}},{\mu\:}_{1U}^{{\uplambda\:}}\right],\left[\sqrt[3]{1-{\left(1-{\vartheta\:}_{1L}^{3}\right)}^{{\uplambda\:}}},\sqrt[3]{1-{\left(1-{\vartheta\:}_{1U}^{3}\right)}^{{\uplambda\:}}}\right]\right)$$

Let $\:{f}_{j}=\left([{\mu\:}_{\mathrm{i}}^{\mathrm{L}},{\mu\:}_{\mathrm{i}}^{U}],\left[{\vartheta\:}_{\mathrm{i}}^{\mathrm{L}}{,\vartheta\:}_{\mathrm{i}}^{\mathrm{U}}\right]\right)\:(i=\mathrm{1,2},\dots\:,\:n)\:$be a collection of IVFFNs, then a “Interval-valued Fermatean fuzzy weighted average (IVFFWA)” is presented as Eq. (5).

5 $$\:IVFFWA=\:\left(\left[\sum\:_{i=1}^{n}{w}_{i}{\mu\:}_{i}^{L},\sum\:_{i=1}^{n}{w}_{i}{\mu\:}_{i}^{U}\right],\:\:\left[\sum\:_{i=1}^{n}{w}_{i}{\vartheta\:}_{i}^{L},\sum\:_{i=1}^{n}{w}_{i}{\vartheta\:}_{i}^{U}\right]\right)$$

where i *= 1*,* 2…*,* n; n* is the number of DMs, and the weight vector $\:w={({w}_{1},{w}_{2},\:\dots\:,{w}_{n})}^{T}\:$is the weight vector of the DMs such that w ∈ [0,1] and $\:\sum\:_{i=1}^{k}{w}_{i}$ =1.

#### Integrated IVFF-PIPRECIA model

This stage determines the relative importance of ergonomic risk criteria under uncertainty. The procedure operationalizes expert linguistic evaluations in an IVFF environment, enabling structured, hesitation-preserving criterion weighting. The weighting process enables the identification of dominant ergonomic risk criteria under explicitly modeled uncertainty. This study uses the IVFF-PIPRECIA methodology to determine the criterion weights. Stanujki´c et al. proposed the fundamental version of the PIPRECIA method^50^. The PIPRECIA method evaluates criteria without requiring a predefined importance ranking, which distinguishes it from many conventional weighting techniques. The method performs pairwise comparisons by referencing the first or last criterion and sequentially evaluating the remaining criteria accordingly. The IVFF-PIPRECIA method integrates IVFF sets with the PIPRECIA technique, combining the strengths of both approaches to enhance decision-making. The procedure of IVFF-PIPRECIA is as follows:

*Step 1. Identification of assessment criteria and decision-makers* Relevant ergonomic risk assessment criteria and decision-makers (DMs) are identified through a comprehensive literature review and expert consultation.

*Step 2. Expert evaluation and criteria weight calculation* DMs individually assess each criterion by comparing it with the first (reference) criterion using the linguistic scale adopted from the literature^51^, as presented in Supplementary Table S2. If a criterion is considered more important than the reference criterion, the corresponding linguistic statement is selected; conversely, if it is considered less important, an appropriate linguistic statement is chosen. For each criterion $\:{C}_{j}$, the relative evaluation provided by the $\:r$-th decision-maker is expressed as.

6 $$\:\stackrel{-}{{h}_{j}^{r}}=\left\{\begin{array}{c}>\stackrel{-}{1}\:\:if\:{C}_{j}>{C}_{j-1\:}\\\:=\stackrel{-}{1}\:\:if\:{C}_{j}={C}_{j-1\:}\\\:<\stackrel{-}{1}\:\:if\:{C}_{j}<{C}_{j-1\:}\end{array}\right.$$

where $\:{\stackrel{\prime }{h}}_{j}^{{\hspace{0.17em}}r}$denotes the relative evaluation of the criterion $\:{C}_{j}$ by decision-maker $\:r$. The selected linguistic statements are subsequently transformed into IVFF numbers using the scale in Table 4. To incorporate the opinions of all decision-makers, the IVFFWA operator (Eq. 5) is applied to aggregate individual evaluations into a collective IVFF evaluation matrix^32^.

The aggregated IVFF evaluations are then converted into crisp values $\:{s}_{j}\:$using the score function. Based on these values, the coefficient $\:{k}_{j}$ (Eq. 7) and the recalculated weight $\:{q}_{j}$are computed as follows (Eq. 8):

7 $$\:{k}_{j}=\left\{\begin{array}{c}=1\:\:\:\:\:\:\:\:\:\:\:\:\:\:\:\:\:\:\:\:\:if\:j=1\\\:2-{s}_{j}\:\:\:\:\:\:\:\:\:\:\:\:\:\:\:\:\:\:if\:\:j>1\end{array}\right.$$

8 $$\:{q}_{j}=\left\{\begin{array}{c}=1\:\:\:\:\:\:\:\:\:\:\:\:\:\:\:\:\:\:\:\:\:\:\:if\:j=1\\\:\frac{{q}_{j-1}}{{k}_{j}}\:\:\:\:\:\:\:\:\:\:\:\:\:\:\:\:\:\:\:\:if\:j>1\end{array}\right.$$

The relative weight of the criterion $\:j$ $\:{(w}_{j}$) is obtained by normalizing the recalculated weights:

9 $$\:{w}_{j}=\frac{{q}_{j}}{\sum\:_{j=1}^{n}{q}_{j}}$$

*Step 3. Inverse evaluation of criteria* To reduce potential bias associated with the selection of the reference criterion, an inverse evaluation is performed. In this step, DMs assess the criteria starting from the last criterion and proceeding backward. The inverse relative evaluation is defined as (Eq. 10).

10 $$\:\stackrel{-}{{h}_{j}^{r{\prime\:}}}=\left\{\begin{array}{c}>\stackrel{-}{1}\:\:if\:{C}_{j}>{C}_{j-1\:}\\\:=\stackrel{-}{1}\:\:if\:{C}_{j}={C}_{j+1\:}\\\:<\stackrel{-}{1}\:\:if\:{C}_{j}<{C}_{j+1\:}\end{array}\right.$$

*Step 4. Final weight aggregation* The final weight of each criterion is determined by averaging the forward and inverse relative weights (Eq. 11).

11 $$\:{w}_{j}^{{\prime\:}{\prime\:}}=\frac{{w}_{j}+\:{w}_{j}^{{\prime\:}}}{2}$$

These final weights serve as the analytical bridge between criterion prioritization and method-level evaluation in the subsequent IVFF–MAIRCA phase.

### Phase 3: weighting of ergonomic risk assessment methods with IVFF-MAIRCA

Building on the criterion weights determined in the previous stage, expert evaluations are transformed into comparative performance scores using the IVFF–MAIRCA method. This process generates a context-sensitive suitability ranking of ergonomic assessment methods, derived from uncertainty-aware weighted criteria. The multi-attributive ideal-real comparative analysis (MAIRCA) is an MCDM method introduced by Gigović et al.54. It evaluates the difference between the theoretical (ideal) solution and the actual (achieved) result. Accordingly, the alternative with the minimum deviation from the ideal solution is considered the most preferable. One of the most notable features of MAIRCA is its impartial treatment of all other options at the outset, giving each an equal opportunity.

Recent studies have demonstrated the effectiveness of integrating an IVFFS into decision-making models to enhance uncertainty representation in complex evaluation problems^52^. Building on this line of research, the present study extends the MAIRCA method by incorporating an IVFFS to evaluate ergonomic risk assessment methods under uncertainty. Accordingly, the proposed IVFF-MAIRCA framework integrates an IVFFS with the MAIRCA technique to assess the relationships between ergonomic risk assessment methods and ergonomic risk criteria. The methodological procedure of the IVFF-MAIRCA approach is outlined below:

*Step 1. Construction of the IVFF decision matrix*. Let $\:{A}_{i}\left(i=\mathrm{1,2},\dots\:,m\right)$ denote the ergonomic risk assessment methods (alternatives), $\:{C}_{j}\left(j=\mathrm{1,2},\dots\:,n\right)$denote the ergonomic risk criteria, and $\:r=\mathrm{1,2},\dots\:,R\:$represent the decision makers.

DMs evaluate each method $\:{A}_{i}$ with respect to the criterion $\:{C}_{j}\:$using the linguistic scale adopted from the literature^53^, as presented in Supplementary Table S3. These linguistic evaluations are transformed into IVFF numbers, forming the individual IVFF decision matrices $\:{\stackrel{\sim}{D}}^{{\hspace{0.17em}}r}$. The aggregated IVFF decision matrix is expressed as (Eq. 12):

12 $$\:{D}^{\sim}=\left[\:{\stackrel{\sim}{x}}_{ij}\right],\:i=1,\dots\:,m,j=1,\dots\:,n$$

where $\:{\stackrel{\sim}{x}}_{ij}$represents the aggregated IVFF evaluation of alternative $\:{A}_{i}$ under criterion $\:{C}_{j}$, obtained by applying the IVFFWA operator to combine the assessments of all decision makers (Eq. 13):

13 $$\:{\stackrel{\sim}{x}}_{ij}=\:IVFFWA({{\stackrel{\sim}{x}}_{ij}}^{1},{{\stackrel{\sim}{x}}_{ij}}^{2},\:\dots\:,\:{{\stackrel{\sim}{x}}_{ij}}^{R})$$

*Step 2. Determination of preference values and theoretical evaluation matrix* In MAIRCA, all alternatives are assumed to have equal initial preferences. Thus, the preference value of each alternative ($\:{P}_{{A}_{i}}$) is defined as (Eq. 14).

14 $$\:{P}_{{A}_{i}}=\frac{1}{m}\:;\sum\:_{i=1}^{m}{P}_{{A}_{i}}=1,\:i=\mathrm{1,2},\dots\:,m$$

Using the criterion weights $\:{w}_{j}$obtained from the IVFF-PIPRECIA method (Sect. "Integrated IVFF-PIPRECIA model"), the theoretical IVFF evaluation matrix ($\:{\stackrel{\sim}{T}}_{{P}_{A}}$) is constructed as Eq. (15).

15 $$\:{\stackrel{\sim}{T}}_{{P}_{A}}\:\:=\left[\:{\stackrel{\sim}{t}}_{{P}_{ij}}\:\right],\:{\:\:\:\:\:\stackrel{\sim}{t}}_{{P}_{\mathrm{i}\mathrm{j}}}=\:\:{P}_{{A}_{\mathrm{i}}}*{w}_{\mathrm{j}}$$

*Step 3. Normalization and construction of the real evaluation matrix* Based on the aggregated IVFF decision matrix $\:{D}^{\sim}\:$obtained in Eq. (12), the normalized initial IVFF decision matrix is first constructed. Let $\:{x}_{j}^{+}$and $\:{x}_{j}^{-}$, which are calculated according to Eq. (16), represent the maximum and minimum values of the criterion $\:{C}_{j}$among all alternatives. The normalized IVFF decision matrix is expressed as $\:\stackrel{\sim}{R}=\left[{\stackrel{\sim}{r}}_{ij}\right]$ .

Each normalized element $\:{\stackrel{\sim}{r}}_{ij}$is calculated via Eq. (16). Here, $\:{J}^{+}$and $\:{J}^{-}$represent the sets of benefit-type and cost-type criteria, respectively.

16 $$\:{\stackrel{\sim}{r}}_{ij}=\left\{\begin{array}{c}\frac{{x}_{ij}-{x}_{j}^{-}}{{x}_{j}^{+}-{x}_{j}^{-}};j\in\:{J}^{+}\\\:\frac{{x}_{ij}-{x}_{j}^{+}}{{x}_{j}^{-}-{x}_{j}^{+}};j\in\:{J}^{-}\end{array}\right.$$

The real evaluation matrix is subsequently constructed by combining the normalized IVFF decision matrix with the theoretical rating matrix derived in Step 2. The elements of the real evaluation matrix $\:{\stackrel{\sim}{T}}_{RA}=\left[{\stackrel{\sim}{t}}_{Rij}\right]\:$are calculated as:

17 $$\:{\stackrel{\sim}{t}}_{{R}_{ij}}=\:\:{\stackrel{\sim}{t}}_{{p}_{ij}}\mathrm{*}{\stackrel{\sim}{r}}_{ij}$$

This matrix presents the actual performance of each ergonomic risk assessment method with respect to each criterion and serves as the basis for the subsequent gap analysis.

*Step 4. Construction of the gap matrix and ranking of alternatives* The IVFF gap matrix $\:{(\:G}^{\sim})$ is obtained by calculating the difference between the theoretical and real evaluation matrices (Eq. 18). The overall gap value $\:{(\:Q}_{i})$ for each alternative is then calculated via Eq. (19)

18 $$\:{G}^{\sim}=\left[\:{\stackrel{\sim}{g}}_{mn}\right],\:\:{g}_{mn}={t}_{{P}_{mn}}-{t}_{{R}_{mn}}$$

19 $$\:{Q}_{i}=\sum\:_{j=1}^{n}{g}_{ij},\:\:i=\mathrm{1,2},\:..,\:m$$

According to the MAIRCA principle, the alternative with the minimum $\:{Q}_{i}$value is considered the most suitable ergonomic risk assessment method.

### Phase 4: determination of the ergonomic risk scores of the operation steps

In this phase, ergonomic risk levels associated with the operational steps of the production process were quantified through the systematic application of six widely used ergonomic risk assessment methods (REBA, RULA, OCRA, QEC, SI, and OWAS) in the enterprise, which strictly followed the original step-by-step scoring procedures defined for each method.

Data collection was conducted through direct workplace observation of representative task cycles performed by operators. During the observations, task-specific parameters required by each method—such as working postures, exerted force/load levels, repetition frequency and duration, and relevant environmental or worker-related factors—were identified and recorded. Based on these inputs, ergonomic risk scores were calculated in accordance with the original scoring logic and scale of each assessment method.

Each assessment method addresses distinct ergonomic dimensions: REBA, RULA, and OWAS focus primarily on posture-related biomechanical load; OCRA and SI quantify repetitive motion and cumulative upper-extremity exposure; and QEC provides a holistic evaluation integrating multiple body regions and perceived discomfort. The combined use of these methods therefore enables comprehensive coverage of ergonomic risk dimensions that may not be fully captured by a single assessment technique.

The ergonomic assessments were conducted on-site by the authors. The resulting method-based scores for all operational steps were organized into a 23 × 6 operation–method decision matrix, as reported in Supplementary Table S4. Raw scores were retained in their original method-specific scales to preserve validated risk thresholds, as the subsequent entropy-based weighting procedure accounts for scale differences through information-based normalization rather than requiring prior score transformation. The detailed case study implementation and numerical results are presented in Sect.  "Case study and numerical results".

### Phase 5: final assessment with Shannon-ENTROPY

In the final stage, the Shannon–ENTROPY method is applied to incorporate objective information dispersion across operational steps. Unlike the preceding expert-driven phases, this stage introduces a data-based weighting mechanism that mitigates subjective bias and enhances analytical robustness. By integrating uncertainty-aware method suitability with objective variability across operational tasks, the framework produces a balanced and structurally consistent final prioritization.

Entropy is a widely used method in MCDM, especially when determining reliable weights based on preferences is challenging or when conducting decision-making experiments is impractical^54^. The technique involves several steps, starting with the normalization ($\:{P}_{ij}$) of the decision matrix, as defined by Eq. (20). Normalization constitutes an essential step of entropy-based weighting procedures, as is commonly applied in MCDM studies^55^.

20 $$\:{P}_{ij}=\frac{{x}_{ij}}{\sum\:_{j=1}^{m}{x}_{ij}}\:\:\:,\:\:\:\forall\:\:ij\:,\:j=\mathrm{1,2},\dots\:,m\:i=\mathrm{1,2},\dots\:,n$$

The weighted normalized decision matrix $\:{M}_{ij}\:$is then formed by multiplying the data in the $\:{P}_{ij}\:$matrix by the corresponding weights as defined in Eq. (21). At this stage, the scores obtained from the IVFF-MAIRCA method are used as weights. The entropy ($\:{E}_{j}$) value is computed via Eq. (22).

21 $$\:{M}_{ij}={P}_{ij}\mathrm{*}\:{w}_{ij}$$

22 $$\:{E}_{j}=\:-\mathrm{k}\:\sum\:_{j=1}^{n}{M}_{ij}\mathrm{ln}\left({M}_{ij}\right)\:\:\:\:\:\:\:\:\:\:\:\:\:\:\:k={\left(\mathrm{ln}\left(n\right)\right)}^{-1}$$

In the given expression, the natural logarithm is denoted “ln” and the constant “k = 1/ln” is obtained from “m” to ensure that the value of $\:\:{E}_{j}$remains in the range [0,1]. Equation (23) denotes the degree of diversification ($\:{D}_{j}$ ). Finally, the relative importance of the attribute is determined as defined in Eq. (24).

23 $$\:{D}_{j}=1-\:{E}_{j},\:\:\:\forall\:\:j$$

24 $$\:{w}_{j}=\:\frac{{D}_{j}}{{\sum\:}_{j=1}^{n}{D}_{j}}\:,\:\:\:\forall\:\:j$$

The methodological framework described above is applied in the following section to an industrial case study, demonstrating its operational applicability and establishing the empirical foundation for addressing the research questions.

## Case study and numerical results

A case study was conducted in a machining company specializing in the production of wagon spare parts to demonstrate the applicability and effectiveness of the proposed ergonomic risk assessment framework. The selected production process involves multiple operational steps, each of which is evaluated for ergonomic risk using the framework described in Sect.  "Materials and methods".

This section presents the implementation of the proposed framework, starting with a description of the production process and operational steps, and followed by the application of the IVFF-PIPRECIA, IVFF-MAIRCA, and ENTROPY methods. The results of the ergonomic risk assessments are analyzed and discussed to provide insights into the most critical ergonomic risks and how they are effectively managed within the production process.

### Production environment and operational steps

The case study was conducted by a company specializing in wagon spare parts. Located in Adana, the company employs over 100 workers and focuses on producing key components such as the 60 kN and 120 kN brake triangles. These products are critical in railway vehicle maintenance and require high-precision manufacturing processes.

The production process includes several operational steps, including hot forging, heat treatment, CNC machining, grinding, and sandblasting. These labor-intensive operations were selected for ergonomic risk assessment due to their significant physical demands and potential impact on worker health and productivity. Each operational step involves distinct ergonomic exposures, such as manual handling of heavy materials, repetitive upper-body movements, and exposure to elevated temperatures. A complete list of the operational steps analyzed in this study is provided in Supplementary Table S5.

Expert opinions and a comprehensive literature review were utilized to systematically evaluate ergonomic risks and identify key factors relevant to these operations. The findings of this study aim to support companies in improving working conditions, mitigating ergonomic risks, and aligning production practices with sustainability objectives, thereby providing a suitable context for demonstrating the applicability of the proposed framework.

### Prioritization of ergonomic risk factors using IVFF-PIPRECIA

This study used the IVFF-PIPRECIA method to prioritize ergonomic risk factors. This method provides an effective tool for prioritizing ergonomic risk factors by objectively combining uncertainty and expert opinion. This methodology provides more accurate results, especially when risk factors involve uncertainty and subjective assessments. Expert evaluations were used to assign weights to each factor, highlighting their relative importance. Tables 3 and 4 show the experts’ assessment of IVFF PIPRECIA.

**Table 3:** Evaluation of criteria by DMs for IVFF PIPRECIA.

Criteria / decision maker	DM1	DM2	DM3
C1	–	–	–
C2	M	SM	MDM
C3	MDM	M	SM
C4	SM	MDM	M
C5	MM	SM	MDM
C6	SM	MDM	M
C7	MDM	M	SM
C8	SM	SM	SM
C9	M	MM	MM
C10	SM	M	MDM
C11	M	M	SM
C12	SM	SM	SM

Linguistic evaluations in this table are based on relative importance expressions (e.g., M = Moderate, SM = Slightly More, MDM = Moderately More, MM = Much More), as defined in the IVFF-PIPRECIA scale (see Supplementary Table S2).

**Table 4:** Evaluation of criteria by DMs for Inverse IVFF PIPRECIA.

Criteria / decision maker	DM1	DM2	DM3
C12	–	–	–
C11	MDL	MDL	WL
C10	MDL	L	MDL
C9	L	RL	MDL
C8	WL	MDL	MDL
C7	WL	MDL	MDM
C6	MDL	L	L
C5	WL	WL	MDM
C4	L	WL	MDM
C3	L	MDL	MDM
C2	MDL	MDL	WL
C1	MDL	WL	MDL

Linguistic terms in this table represent inverse comparison judgments (e.g.,MDL = Moderately Less, WL = Weakly Less,RL = Relatively Less), as defined in the inverse IVFF-PIPRECIA scale (see Supplementary Table S2)

**Fig. 2:**
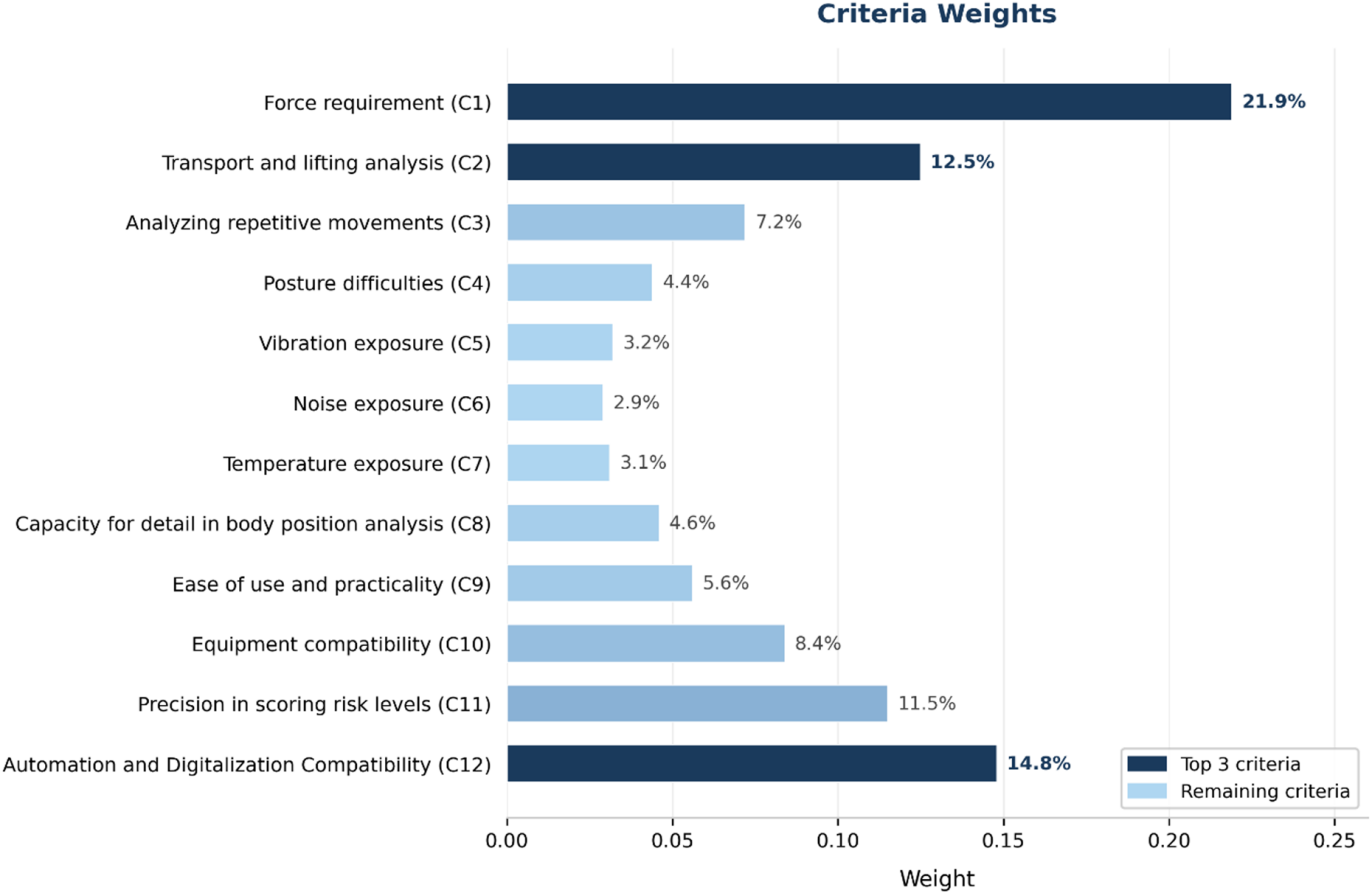
Criteria weights for ranking ergonomic risk assessment alternatives. This figure shows the final criterion weights obtained using the IVFF-PIPRECIA method, highlighting the relative importance of ergonomic risk factors considered in the evaluation.

On the basis of the expert evaluations presented in Tables 3 and 4, the IVFF-PIPRECIA procedure defined in Eqs. (6–11) was applied to derive the final criterion weights. The resulting aggregated weights, which constitute the basis for subsequent method ranking, are illustrated in Fig. 2.

Figure 2 shows that *force requirement (C1) and automation and digitalization compatibility (C12)* are the most influential criteria, with weights of *21.9% and 14.8%*, respectively. *Transport and lifting analysis (C2)* and *precision in scoring risk levels (C11)* show moderate significance at *12.4%* and *11.5%*, respectively. In contrast, criteria such as *vibration exposure (C5)*,* temperature exposure (C7)*, and *noise exposure (C6*) have minimal impact, with weights below *4%*, underscoring the prioritization of physical and technological aspects over environmental considerations.

### Weighting ergonomic risk assessment methods using IVFF-MAIRCA

This section discusses integrated approaches to risk assessment based on ergonomic methods using IVFF and the MAIRCA method. This analysis assesses and ranks various ergonomic risk assessment tools based on multiple criteria relevant to accurate and reliable ergonomic risk evaluation. The selected methods include REBA, RULA, OCRA, QEC, SI, and OWAS; the effectiveness to be considered will regard the assessment of ergonomic risk factors, namely force requirements, posture difficulties, repetitive motions, and other conditions. The IVFF thus permits modeling uncertainty and subjectivity that may characterize expert judgments, an important feature when high-precision numerical values are unavailable. A MAIRCA will provide a much broader and more complete view of its usability across different tasks within the manufacturing environment by establishing a comprehensive, structured approach to deriving the weight of ergonomic risk assessment methods.

In this framework, the IVFF-PIPRECIA method is employed to determine the importance weights of ergonomic risk factors, such as *C1 (force requirements)*,* C2(transport and lifting analysis)*, and *c3 (repetitive motions)*, among others. For example, the IVFF-PIPRECIA analysis revealed that *C1 (force requirements)* has the highest weight ($\:{w}_{{C}_{1}}=0.219$), indicating its critical role in ergonomic risk evaluation. Similarly, *C3(repetitive motions)* and *C4 (posture difficulties)* were assigned weights of ($\:{w}_{{C}_{3}}=0.072,\:and\:{w}_{{C}_{4}}=0.044)\:$, emphasizing their moderate significance in the assessment process. These weights were then utilized in the IVFF-MAIRCA method to evaluate and rank the selected ergonomic risk assessment methods.

Within the IVFF-MAIRCA framework, expert judgments regarding the performance of ergonomic risk assessment methods with respect to the prioritized criteria are first expressed using IVFF linguistic terms. Table 5 presents the linguistic evaluations provided by the decision makers, reflecting their collective perceptions of each method’s adequacy with respect to the considered ergonomic criteria.

These linguistic assessments are subsequently transformed and processed following the IVFF-MAIRCA computational procedure to obtain the final gap values for each alternative. The resulting total gaps, which represent the discrepancy between the theoretical (ideal) and actual performance of each method, are reported in Table 6. According to the MAIRCA principle, ergonomic risk assessment methods with smaller total gap values are considered more suitable.

**Table 5:** Linguistic assessment of ergonomic risk assessment methods by experts.

Alternatives	Decision makers	Criteria											
		C1	C2	C3	C4	C5	C6	C7	C8	C9	C10	C11	C12
EAM1	DM1	M	H	MH	H	H	ML	VH	L	M	MH	MH	L
	DM2	MH	H	H	VH	MH	M	H	ML	MH	H	H	L
	DM3	H	VH	H	H	VH	ML	H	L	MH	VH	H	ML
EAM2	DM1	ML	VH	H	MH	MH	M	VH	L	MH	MH	MH	L
	DM2	MH	VH	H	MH	H	ML	VH	ML	MH	H	H	ML
	DM3	M	H	VH	H	VH	ML	H	L	H	VH	H	ML
EAM3	DM1	MH	VH	VH	H	VH	ML	VH	L	MH	MH	MH	ML
	DM2	H	VH	VH	H	VH	ML	VH	ML	H	MH	H	ML
	DM3	MH	VH	VH	H	H	ML	VH	L	H	MH	MH	ML
EAM4	DM1	M	H	H	MH	H	ML	MH	L	M	MH	MH	ML
	DM2	MH	H	H	MH	MH	ML	H	ML	M	MH	MH	ML
	DM3	M	MH	MH	MH	MH	ML	H	L	MH	H	H	ML
EAM5	DM1	MH	H	H	H	MH	M	MH	ML	MH	H	MH	ML
	DM2	H	VH	VH	H	H	M	H	ML	MH	VH	MH	L
	DM3	MH	VH	H	H	H	ML	H	L	MH	VH	H	ML
EAM6	DM1	ML	M	MH	MH	H	ML	MH	ML	MH	M	MH	ML
	DM2	M	MH	M	M	MH	L	H	ML	M	MH	MH	ML
	DM3	ML	MH	M	MH	MH	L	H	ML	M	MH	MH	L

Linguistic terms represent risk levels using a standardized scale, where VL = Very Low, L = Low, ML = Medium-Low, M = Medium, MH = Medium-High, H = High, and VH = Very High. The corresponding IVFF representations are provided in Supplementary Table S3.)

**Table 6:** Total gaps for the alternatives of IVFF – MAIRCA.

Alternatives	EAM1	EAM2	EAM3	EAM4	EAM5	EAM6
$\:{\boldsymbol{Q}}_{\boldsymbol{i}}$	0,101	0,086	0,096	0,082	0,116	0,072

Table 6 presents the total gaps ($\:{Q}_{i}$) for each ergonomic assessment method evaluated using the IVFF-MAIRCA method. Within the MAIRCA framework, lower $\:{Q}_{i}$values indicate a closer alignment between the theoretical (ideal) and actual performance profiles of an alternative with respect to the prioritized ergonomic criteria.

The results show that *EAM6 (SI)*,* EAM4 (OWAS)*, and *EAM2 (RULA)* exhibit comparatively lower total gap values, indicating that these methods provide a more balanced and consistent performance across the evaluated ergonomic criteria and are therefore more suitable for assessing ergonomic risks in the operational steps considered. In contrast, *EAM5 (OCRA)* yields the highest total gap value ($\:{Q}_{i}=0.116$), reflecting a larger deviation from the ideal performance profile. This suggests that, under the current criteria weighting structure, OCRA is less well aligned with the prioritized ergonomic requirements than the other evaluated methods.

### Final assessment using Shannon-ENTROPY

In the final stage of the analysis, the Shannon-ENTROPY method was employed to derive objective importance weights for the operational steps within the production process. Unlike the preceding IVFF-PIPRECIA and IVFF-MAIRCA stages, which incorporate expert judgments, the ENTROPY method relies solely on the inherent information content of the decision matrix, thereby providing an objective complement to the subjective evaluations.

The ergonomic risk scores obtained for the operational steps using six different assessment methods constitute the primary input for the entropy-based evaluation. These method-specific scores, reported in Supplementary Table S4, are combined with the method suitability weights obtained from the preceding analyses to enable objective prioritization of operational ergonomic risks.

The ENTROPY analysis was conducted using the initial decision matrix presented in Table 7. Following normalization (Eq. 20), the entropy values $\:{e}_{j}$ were calculated using Eq. (22). Given the presence of six alternatives, the entropy constant was computed as $\:k=({\mathrm{ln}\left(6\right))}^{-1}=\mathrm{0,5581}$. The degrees of diversification $\:{d}_{j}\:$(Eq. 23) and the corresponding entropy weights $\:{w}_{j\:}$(Eq. 24) were subsequently obtained.

Table 8 reports the calculated $\:{e}_{j}$, $\:{d}_{j}$, and $\:{w}_{j}$values for all the operational steps. Higher entropy weights indicate operational steps with greater variability and informational significance across the evaluated ergonomic assessment methods and therefore represent a higher ergonomic risk priority.

**Table 7:** Initial decision matrix.

	O1	O2	O3	O4	O5	O6	O7	O8	O9	O10	O11	O12
EAM1	6,00	4,00	5,00	5,00	4,00	6,00	4,00	7,00	5,00	5,00	6,00	4,00
EAM2	5,00	4,00	4,00	6,00	4,00	5,00	4,00	6,00	5,00	6,00	5,00	4,00
EAM3	69,14	70,37	72,22	70,37	71,60	72,22	70,37	65,43	70,37	77,16	65,43	77,16
EAM4	2,00	3,00	3,00	3,00	3,00	2,00	2,00	4,00	3,00	3,00	4,00	3,00
EAM5	10,00	9,00	8,00	12,00	10,00	8,00	9,00	11,00	10,00	13,00	12,00	9,00
EAM6	10,00	9,00	10,00	10,00	9,00	9,00	9,00	10,00	9,00	10,00	10,00	9,00

	O13	O14	O15	O16	O17	O18	O19	O20	O21	O22	O23
EAM1	7,00	6,00	6,00	6,00	4,00	5,00	5,00	4,00	5,00	6,00	5,00
EAM2	6,00	5,00	5,00	5,00	4,00	5,00	6,00	5,00	5,00	6,00	5,00
EAM3	76,54	70,37	70,37	71,60	70,37	72,22	70,37	76,54	70,37	74,69	65,43
EAM4	4,00	3,00	3,00	4,00	3,00	3,00	3,00	3,00	2,0	3,00	2,00
EAM5	12,00	10,00	10,00	12,00	9,00	10,00	12,00	11,00	10,00	12,00	9,00
EAM6	10,00	10,00	9,00	10,00	9,00	10,00	10,00	10,00	9,00	10,00	9,00

**Table 8:** $\:{e}_{j}$, $\:{d}_{j}$ and $\:{w}_{j}$ values of the decision criteria.

	$\:{\boldsymbol{e}}_{\boldsymbol{j}}$	$\:{\boldsymbol{d}}_{\boldsymbol{j}}$	$\:{\boldsymbol{w}}_{\boldsymbol{j}}$		$\:{\boldsymbol{e}}_{\boldsymbol{j}}$	$\:{\boldsymbol{d}}_{\boldsymbol{j}}$	$\:{\boldsymbol{w}}_{\boldsymbol{j}}$		$\:{\boldsymbol{e}}_{\boldsymbol{j}}$	$\:{\boldsymbol{d}}_{\boldsymbol{j}}$	$\:{\boldsymbol{w}}_{\boldsymbol{j}}$
O1	0,6197	0,3803	0,0207	**O9**	0,1818	0,8182	0,0445	**O17**	0,1786	0,8214	0,0447
O2	0,1786	0,8214	0,0447	**O10**	0,1831	0,8169	0,0445	**O18**	0,1814	0,8186	0,0445
O3	0,1785	0,8215	0,0447	**O11**	0,1875	0,8125	0,0442	**O19**	0,1846	0,8154	0,0444
O4	0,1846	0,8154	0,0444	**O12**	0,1763	0,8237	0,0448	**O20**	0,1797	0,8203	0,0446
O5	0,1792	0,8208	0,0447	**O13**	0,1852	0,8148	0,0443	**O21**	0,1818	0,8182	0,0445
O6	0,1790	0,8210	0,0447	**O14**	0,1831	0,8169	0,0444	**O22**	0,1841	0,8159	0,0444
O7	0,1775	0,8225	0,0448	**O15**	0,1828	0,8172	0,0445	**O23**	0,1815	0,8185	0,0445
O8	0,1884	0,8116	0,0442	**O16**	0,1853	0,8147	0,0443				

Based on the entropy weights, the operational steps were ranked as follows:

O12 >> O7 >> O3 >> O2 >> O17 >> O6 >> O5 >> O20 >> O18 >> O23 > > O9 >> O21 >> O15 >> O10  >> O14 >> O22 >> O4 >> O19 >> O13 >> O16 >> O11 >> O8 >> O1.

**Fig. 3:**
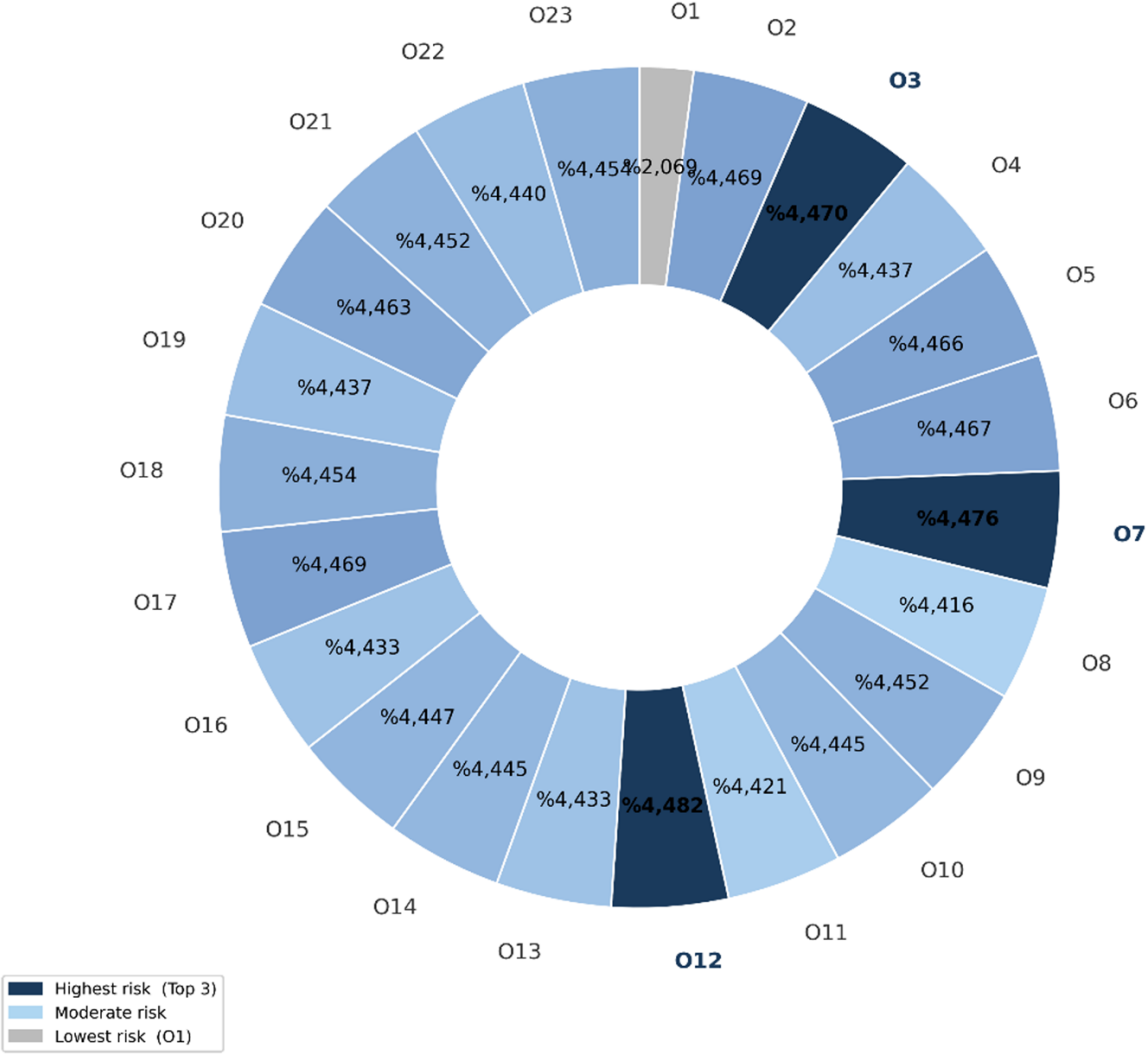
Ergonomic risk levels for operations. This figure visualizes the entropy-based ergonomic risk levels for each operational step, indicating which operations have a higher ergonomic priority for intervention.

Figure 3 presents a visual representation of these entropy-based weights, showing the relative ergonomic risk levels for each operational step. Operations with higher weights, such as *O12 (quality control of body draft)* and *O7 (quality control of procured raw material for holders)*, emerge as the most critical stages from an ergonomic perspective. These tasks are characterized by intensive inspection activities and the handling of heavy, mechanized components, which result in elevated ergonomic loads. In contrast, operations with lower weights, such as *O1* and *O8*, exhibit comparatively lower ergonomic risk and therefore require less immediate intervention.

Overall, integrating IVFF-MAIRCA scores as alternative weights with ENTROPY-based operational prioritization enables a comprehensive assessment framework that combines subjective expert knowledge with objective, data-driven insights. This final evaluation stage supports decision-makers in identifying the most ergonomically critical operational steps and prioritizing targeted risk mitigation strategies.

## Comparative and robustness analysis

This section examines how alternative fuzzy information structures influence both criterion weighting and alternative ranking outcomes within the proposed framework. By maintaining identical expert evaluations and linguistic scales while varying the fuzzy representation (IF, PF, and IVFF), the analysis isolates the structural effect of uncertainty modeling. In addition, robustness tests are conducted to assess the stability of ranking outcomes under controlled perturbations.

### Impact of fuzzy framework selection on criteria weights

To examine the effect of fuzzy information representation on criterion weighting, IF-PIPRECIA, PF-PIPRECIA, and IVFF-PIPRECIA were applied using the same set of criteria, identical expert panels, and consistent methodological procedures. The only variation across the three implementations lies in the mathematical structure of the fuzzy sets used to express expert judgments.

Table 9 reports the normalized criterion weights obtained under each fuzzy framework. The results indicate both stable and variable weighting patterns across the three approaches. A rank reversal is observed between C1 and C12: while IF-PIPRECIA and PF-PIPRECIA assign the highest weight to C12, IVFF-PIPRECIA identifies C1 as the most influential criterion, with C12 ranked second.

**Table 9:** Comparison of criterion weights across fuzzy frameworks.

Criterion	IF-PIPRECIA	PF-PIPRECIA	IVFF-PIPRECIA	Mean	Std. Dev.	Rank range
C1	0.210	0.207	0.219	0.212	0.0063	1–2
C12	0.272	0.277	0.148	0.232	0.0742	1–2
C2	0.122	0.121	0.125	0.123	0.0021	3–4
C11	0.126	0.126	0.115	0.122	0.0063	3–4
C3	0.071	0.071	0.072	0.071	0.0006	5–6
C10	0.058	0.057	0.084	0.066	0.0153	5–6
C4	0.042	0.042	0.044	0.043	0.0012	7–9
C9	0.027	0.026	0.056	0.036	0.0171	7–8
C5	0.025	0.026	0.032	0.028	0.0038	9–10
C8	0.016	0.015	0.046	0.026	0.0175	8–11
C6	0.016	0.017	0.029	0.021	0.0073	10–12
C7	0.014	0.014	0.031	0.020	0.0098	11–12

For criteria with relatively high importance (C1, C2, C3, and C11), numerical differences across frameworks remain limited, with standard deviations below 0.007. In contrast, several mid-ranked criteria, including C12, C9, C8, and C10, exhibit noticeable sensitivity to the employed fuzzy representation, as reflected in larger numerical deviations and wider rank ranges.

The lower-weighted criteria (e.g., C4 and C5) maintain consistent positions across all three frameworks, with rank ranges confined to two or three positions and minimal numerical variation. The overall dispersion characteristics differ across methods, as summarized by the coefficient of variation values reported in Table 9.

**Fig. 4:**
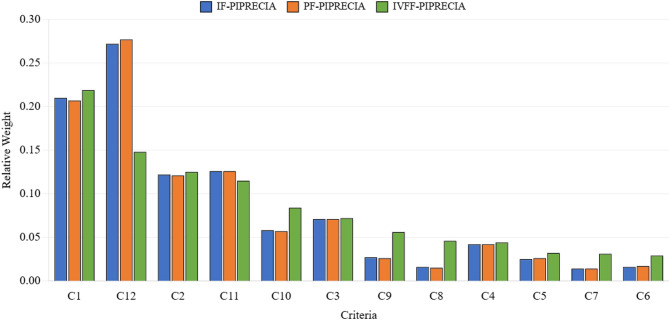
Criterion weights comparison. This figure compares the criterion weight distributions obtained under intuitionistic fuzzy, Pythagorean fuzzy, and interval-valued Fermatean fuzzy environments.

Figure 4 visualizes the distribution of criterion weights across IF-, PF-, and IVFF-based implementations, highlighting differences in the concentration of weights among the three fuzzy representations.

Having examined the impact of fuzzy representation on criterion weighting, the analysis now turns to the implications of these differences for alternative ranking outcomes.

### Impact of fuzzy framework selection on alternative rankings

IF-MAIRCA, PF-MAIRCA, and IVFF-MAIRCA were applied using the criterion weights obtained in Sect. "Impact of fuzzy framework selection on criteria weights". The same set of ergonomic assessment methods (EAM1–EAM6) and identical computational steps were employed across all three frameworks. In the MAIRCA method, lower $\:Q$ values indicate superior performance.

**Table 10:** Comparison of MAIRCA results under different fuzzy frameworks.

Alternative	IF-MAIRCA Q	Rank	PF-MAIRCA Q	Rank	IVFF-MAIRCA Q	Rank
EAM1	0.1107	6	0.1144	6	0.1011	5
EAM2	0.0705	3	0.0730	3	0.0865	3
EAM3	0.0987	4	0.0976	4	0.0957	4
EAM4	0.0506	1	0.0514	1	0.0821	2
EAM5	0.1018	5	0.1050	5	0.1160	6
EAM6	0.0589	2	0.0596	2	0.0722	1

Rank = 1 denotes the most preferable alternative.

Table 10 reports the $\:Q$ values and corresponding rankings for each alternative under the three fuzzy frameworks. IF-MAIRCA and PF-MAIRCA yield identical rankings, placing EAM4 in the first position ($\:Q=0.0506$ and $\:0.0514$, respectively), followed by EAM6 in the second position. Under IVFF-MAIRCA, this ordering is reversed, with EAM6 ranked first ($\:Q=0.0722$) and EAM4 second ($\:Q=0.0821$).

Mid-ranked alternatives exhibit complete positional stability. EAM2 and EAM3 retain the third and fourth ranks, respectively, across all three frameworks. The lower-ranked alternatives show limited variation: EAM5 ranks fifth in IF-MAIRCA and PF-MAIRCA, but sixth in IVFF-MAIRCA, whereas EAM1 shifts from sixth in IF/PF to fifth in IVFF.

The Spearman rank correlation coefficients between frameworks are as follows: ρ (IF, PF) = 1.000, ρ (IVFF, IF) = 0.886, and ρ (IVFF, PF) = 0.886.

**Fig. 5:**
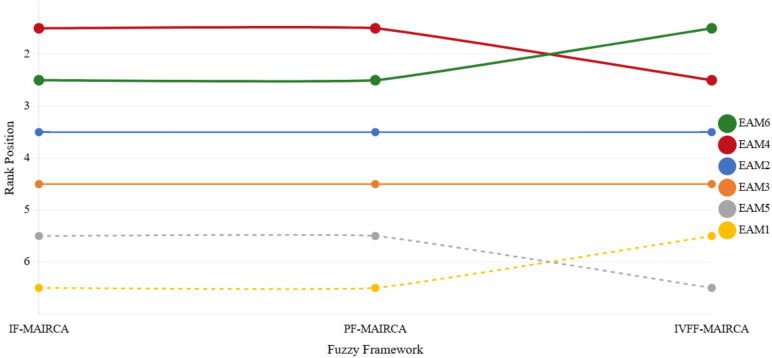
Alternative rank trajectories across fuzzy frameworks. This figure illustrates the ranking trajectories of ergonomic risk assessment methods across different fuzzy modeling frameworks, highlighting changes in relative performance under varying uncertainty representations.

Figure 5 illustrates the rank trajectories of all alternatives across the three fuzzy frameworks, showing the position exchange between EAM4 and EAM6 and the consistent rankings of EAM2 and EAM3.

Beyond methodological robustness, the comparative results provide decision-relevant insights for industrial practice. The stability analysis demonstrates that the proposed IVFF-based structure maintains ranking coherence while refining discrimination near decision-critical positions. For practitioners, this implies that ergonomic assessment method selection can be based on structured suitability analysis rather than habitual preference, and that intervention priorities across operational steps can be determined through transparent, data-supported reasoning. In practical terms, managers can use the framework outputs to allocate ergonomic improvement resources toward the most critical operations and to justify method selection decisions within safety and production planning processes. The framework therefore supports not only analytical consistency but also more accountable, defensible ergonomic risk management in manufacturing environments.

While the preceding comparison evaluates structural differences across fuzzy representations, it remains necessary to verify whether the proposed IVFF-based ranking remains stable under controlled variations in linguistic interval definitions. Therefore, a robustness analysis is conducted in the following subsection.

### Robustness analysis

To examine whether the final decision outcomes are sensitive to the definition of linguistic membership intervals — a critical modeling component in expert-based IVFF evaluations — a robustness analysis was conducted using proportional interval-width perturbation (*k* ∈ {0.9, 1.0, 1.1}). Here, *k = 1.0* represents the original linguistic definitions (base case), while *k = 0.9* and *k = 1.1* correspond to 10% contraction and expansion of interval widths, respectively. Central tendencies were preserved to maintain semantic consistency, and all modified intervals satisfied the IVFF feasibility constraint.

For each scenario, the entire IVFF–PIPRECIA–MAIRCA–ENTROPY pipeline was fully re-executed. Criterion weights were recalculated under IVFF–PIPRECIA, updated weights were used in IVFF–MAIRCA to derive method suitability scores, and the Shannon–ENTROPY stage was subsequently applied to obtain final operational risk rankings.

The ranking of the six ergonomic assessment methods remained identical across all scenarios (*Spearman ρ = 1.000; 0/15 pairwise rank reversals)*, indicating complete stability of method prioritization under moderate linguistic variation. A correlation coefficient of 1.000 indicates perfect agreement between the compared ranking structures.

At the operational prioritization level, the ENTROPY-based aggregation was recomputed using the updated suitability weights. The ranking of the 23 operational steps also remained unchanged (*Spearman ρ = 1.000; 0 rank reversals).* Minor numerical differences were observed in intermediate weights; however, these variations were insufficient to alter the final ordering.

These results confirm the structural robustness of the entire IVFF–PIPRECIA–MAIRCA–ENTROPY decision architecture under controlled linguistic interval perturbations, as summarized in Table 11.

**Table 11:** Spearman rank correlations and reversal count under interval-width perturbation (base: *k = 1.0*).

Scenario	k	(Methods) ρ	(Operations) ρ	Rank Reversals
Contracted	0.9	1.000	1.000	0
Expanded	1.1	1.000	1.000	0

*Note: Spearman correlations are calculated relative to the base case (k = 1.0).*

The following section discusses these findings explicitly in relation to the three research questions formulated in Sect. "Research gaps and contributions".

## Discussion

This section interprets the empirical results presented in Sect. "Comparative and robustness analysis" in relation to the three research questions, with particular emphasis on how uncertainty-aware modeling and multi-criteria integration influence ergonomic risk assessment outcomes.

### Influential ergonomic risk factors under uncertainty

This subsection addresses RQ1, which seeks to identify the ergonomic risk factors that are most influential when expert judgments are evaluated under uncertainty. The analysis builds directly on the IVFF-PIPRECIA weighting results presented in Sect. "Impact of fuzzy framework selection on criteria weights", where criterion importance was derived using identical expert panels and decision structures across different fuzzy representations.

Across all fuzzy representations, criteria related to force requirements (C1) and automation compatibility (C12) consistently appear among the most influential factors. These criteria capture fundamental characteristics of the evaluated machining environment, where manual material handling, physically demanding operations, and limited automation substantially increase MSD risk^9,12^. The prominence of these factors suggests that ergonomic risk is driven primarily by tasks involving high physical load and insufficient technological support, rather than by many moderately essential factors.

The relatively high weight assigned to automation compatibility (C12) reflects the ongoing digital transformation of contemporary manufacturing systems, particularly in environments where sensor-based monitoring, data-driven control architectures, and advanced human–machine interaction technologies are progressively integrated into production processes. In such contexts, ergonomic assessment methods that can interface with digital infrastructures acquire enhanced strategic relevance. The expert evaluations, therefore, capture not only current operational requirements but also reflect forward-looking technological adaptability within evolving industrial settings. Accordingly, the elevated position of C12 within the weighting structure represents a decision-analytic and normative prioritization of strategic compatibility, rather than a direct empirical quantification of technology adoption levels.

In contrast, several criteria associated with posture variation, repetition, and environmental conditions exhibit intermediate levels of importance. The relative influence of the factors varies across operational contexts and task characteristics, suggesting that these factors contribute to ergonomic risk in a more situation-dependent manner. The lower-ranked criteria, including those related to secondary physical or organizational aspects, consistently have a limited impact on the overall weighting structure, regardless of the uncertainty modeling approach.

These interpretations are grounded in the IVFF-PIPRECIA weighting structure presented in Sect. "Impact of fuzzy framework selection on criteria weights", ensuring that the discussion reflects the uncertainty-aware aggregation of expert judgments. Overall, the results demonstrate that when uncertainty is explicitly considered, ergonomic risk assessment highlights a concentrated importance structure dominated by a small number of critical factors. From a practical perspective, this finding implies that ergonomic improvement strategies should prioritize high-load, low-automation operations, as addressing these dominant risk factors is likely to yield the most significant reduction in overall ergonomic risk.

### Comparative performance of the ergonomic assessment methods

This subsection responds to RQ2 by examining how traditional ergonomic risk assessment methods perform when systematically analyzed within a unified uncertainty-aware fuzzy MCDM framework.

In interpreting these results, it is necessary to clearly delineate the scope of the proposed framework. The study does not aim to clinically validate associations with musculoskeletal disorder (MSD) incidence. Instead, the framework is designed as a methodological decision-support tool for the comparative prioritization of ergonomic risk assessment methods under uncertainty. Its contribution lies in enhancing methodological consistency, structured evaluation, and comparative transparency, rather than establishing epidemiological relationships between assessment outcomes and worker health data. The comparison does not imply methodological superiority in absolute terms; rather, it reflects relative suitability within the defined industrial context and evaluation structure.

Table 12 presents the comparative results for 23 manufacturing operations. Notable discrepancies emerge between individual method outcomes and the integrated framework. Task O1 receives high criticality scores from REBA (6) and SI (10) because of their emphasis on postural strain and biomechanical load. However, under the proposed framework—which simultaneously considers repetition, environmental conditions, and task variability—O1’s relative priority decreases to 2.499%. This demonstrates how single-method assessments may overstate risk when focused on a limited set of ergonomic dimensions.

Conversely, operation O12 emerges as the most critical task according to the proposed method (4.510%), despite receiving only moderate scores from individual techniques (REBA: 4, RULA: 4). This outcome reflects the cumulative effect of multiple risk dimensions weighted according to their integrated importance, altering risk prioritization relative to traditional single-method assessments. These interpretations are grounded in the IVFF-PIPRECIA weighting structure presented in Sect. "Impact of fuzzy framework selection on criteria weights", ensuring that the discussion reflects the uncertainty-aware aggregation of expert judgments.

Overall, these results demonstrate that systematic comparison within a unified fuzzy MCDM framework provides deeper insights into method performance and supports more balanced ergonomic decision-making, which is consistent with recent multimethod evaluation studies^56,57^. Unlike most existing ergonomic assessment studies, which rely on single-method evaluations, the present study demonstrates that an integrated fuzzy MCDM analysis can substantially alter task prioritization by accounting for cumulative risk across multiple ergonomic dimensions.

**Table 12:** Comparison of the results.

Operations	REBA score	RULA score	QEC score	OWAS score	OCRA score	SI score	Proposed Method (%)
O1	**6**	5	69.14	2	10	**10**	2.499
O2	4	4	70.37	3	9	9	**4.468**
O3	5	4	72.22	3	8	**10**	**4.470**
O4	5	**6**	70.37	3	**12**	**10**	4.398
O5	4	4	71.6	3	10	9	**4.467**
O6	**6**	5	72.22	2	8	9	**4.519**
O7	4	4	70.37	2	9	9	**4.535**
O8	**7**	**6**	65.43	**4**	11	**10**	4.300
O9	5	5	70.37	3	10	9	4.433
O10	5	**6**	**77.16**	3	**13**	**10**	4.432
O11	**6**	5	65.43	**4**	**12**	**10**	4.313
O12	4	4	**77.16**	3	9	9	**4.510**
O13	**7**	**6**	**76.54**	**4**	**12**	**10**	4.366
O14	**6**	5	70.37	3	10	**10**	4.416
O15	**6**	5	70.37	3	10	9	4.424
O16	**6**	5	71.6	**4**	**12**	**10**	4.355
O17	4	4	70.37	3	9	9	**4.468**
O18	5	5	72.22	3	10	**10**	4.437
O19	5	**6**	70.37	3	**12**	**10**	4.398
O20	4	5	**76.54**	3	11	**10**	**4.466**
O21	5	5	70.37	3	10	9	4.433
O22	**6**	**6**	**74.69**	3	**12**	**10**	4.415
O23	5	5	65.43	2	9	9	**4.476**

### Advantages of IVFFS over other fuzzy approaches

This subsection directly addresses RQ3 by examining how the enhanced representational capacity of IVFFS influences both weighting stability and ranking sensitivity. A key methodological contribution of this study lies in the controlled comparison of intuitionistic fuzzy (IF), Pythagorean fuzzy (PF), and interval-valued Fermatean fuzzy (IVFF) environments within an identical decision-making structure. Unlike IF and PF sets, which represent expert judgments using single-valued membership and non-membership degrees, IVFFS models evaluations as bounded intervals under a Fermatean constraint. This approach preserves hesitation and ambiguity throughout the aggregation and weighting processes. This extended representational space enables a richer description of uncertainty, which has been identified as a critical requirement in expert-based decision-making contexts, including ergonomic risk assessment^49^.

The advantages of IVFFS become evident at the criterion weighting stage. As demonstrated in Sect. "Impact of fuzzy framework selection on criteria weights", IVFF-PIPRECIA produces a more balanced distribution of criterion weights than IF- and PF-based implementations do, as reflected in a lower coefficient of variation. While dominant criteria remain clearly identifiable across all frameworks, IVFFS enhances discrimination among mid-ranked criteria, which tend to be compressed into narrow importance ranges under single-valued fuzzy representations. This behavior indicates that IVFFS refines the importance structure by preserving uncertainty-induced variability rather than suppressing it through point-based approximations.

At the alternative ranking stage, the impact of the IVFFS is further accentuated. Section "Impact of fuzzy framework selection on alternative rankings" shows that IF-MAIRCA and PF-MAIRCA yield identical rankings, whereas IVFF-MAIRCA introduces a rank reversal between the two top-performing ergonomic assessment methods. Notably, this change occurs at decision-critical positions while maintaining a high overall rank correlation (ρ = 0.886), indicating that the interval-valued framework increases sensitivity near the optimum without destabilizing the global ranking structure. Such controlled refinement is particularly important in method selection problems, where small performance differences may have significant practical implications. This finding differs from prior studies that employed a single fuzzy framework, showing that the choice of fuzzy representation can influence decision-critical ranking outcomes, even when all other modeling components are held constant.

From an ergonomic decision-making perspective, IVFFS offers an additional advantage by explicitly signaling residual uncertainty to decision-makers. The proximity of the Q values between the top-ranked alternatives under IVFF-MAIRCA suggests that superiority is contingent rather than absolute, encouraging cautious interpretation when expert consensus is limited. In contrast, the IF and PF frameworks collapse interval information into single values, potentially conveying a false sense of certainty. Preserving this ambiguity is especially valuable in ergonomic risk assessment, where oversimplified rankings may lead to suboptimal method selection and implementation decisions^18,19.^

Overall, these findings demonstrate that IVFFS improves uncertainty modeling not by altering the underlying decision logic, but by enriching the information structure on which that logic operates, thereby directly addressing RQ3.

Importantly, this enhanced representational capacity does not introduce additional computational burden. In the presented case study involving 12 ergonomic risk factors and 6 assessment methods, each expert completed 2 structured evaluation matrices for criteria weighting and alternative assessment. The framework, therefore, remains practically manageable for real-world decision-making contexts.

Furthermore, as contemporary research increasingly emphasizes real-time ergonomic monitoring through wearable sensors and AI-driven predictive systems in digitally integrated manufacturing environments, the proposed framework offers a complementary methodological perspective. While sensor-based approaches primarily focus on dynamic risk detection and real-time feedback, the IVFF–MCDM model contributes structured decision support for prioritizing and selecting appropriate ergonomic assessment methods under uncertainty. In this way, the framework supports systematic methodological decision-making within evolving digital industrial contexts.

From an industrial implementation perspective, the proposed framework provides a structured decision pathway for ergonomic risk management in complex manufacturing environments. Decision-makers can first identify dominant ergonomic risk factors under uncertainty through the derived criterion weights, then select the most appropriate ergonomic assessment method based on the obtained method rankings rather than subjective familiarity and finally prioritize operational steps for targeted intervention planning using the aggregated risk results. This staged decision logic reduces reliance on subjective judgment and supports more transparent, consistent, and defensible ergonomic decision-making in practice.

## Limitations and future research directions

While this study proposes an IVFFS-based integrated framework and demonstrates its applicability through a real-world manufacturing case, several limitations should be acknowledged. First, the evaluation relies on judgments from three expert decision makers. Although these experts possess substantial domain knowledge, future studies could benefit from larger and more heterogeneous expert panels to further increase robustness and reduce potential interrater bias.

Second, the empirical analysis is conducted within a single manufacturing facility. While this case study provides valuable insights into practical implementation, extending the analysis to multiple production environments would strengthen external validity and enable broader generalization of the findings.

Third, the combined use of IVFF-PIPRECIA, IVFF-MAIRCA, and ENTROPY enables comprehensive uncertainty modeling but may introduce computational and conceptual complexity for practitioners unfamiliar with fuzzy MCDM techniques. Future research could address this challenge by developing decision-support software or simplified interfaces that facilitate practical adoption without compromising methodological rigor.

In this context, future studies may focus on (i) incorporating experts from diverse industrial sectors, (ii) validating the framework across multiple manufacturing settings, and (iii) exploring alternative uncertainty modeling paradigms, such as hesitant or neutrosophic fuzzy sets, to further enhance decision robustness, and (iv) integrating participatory ergonomics inputs, such as worker discomfort reports and subjective perceptions, as explicit decision criteria within the framework.

## Conclusion

The present study proposes an integrated ergonomic risk assessment framework designed to support systematic and uncertainty-aware decision-making in manufacturing environments. The primary scientific contribution of this study lies in the joint implementation of IVFF - PIPRECIA, IVFF-MAIRCA, and Shannon’s Entropy method within a unified structure that simultaneously addresses subjective expert judgments and objective operational data. This hybrid design extends existing ergonomic assessment methodologies by explicitly incorporating interval-valued Fermatean fuzzy logic. This incorporation enhances the representation of epistemic uncertainty and improves discrimination power in multi-criteria evaluations.

From a methodological perspective, the comparative analysis across different fuzzy environments demonstrated that the IVFF structure achieves more refined differentiation among decision-critical criteria and alternatives while preserving ranking stability with respect to conventional fuzzy extensions. This finding confirms that the proposed framework does not merely increase model complexity but provides tangible analytical advantages in resolving closely competing ergonomic risk factors and assessment methods.

In practical terms, the framework offers a transparent and reproducible decision-support tool for manufacturing practitioners. It facilitates the systematic prioritization of ergonomic risk factors, the rational selection of assessment methods based on structured suitability analysis rather than habitual preference, and the objective ranking of operational steps requiring intervention. Consequently, the proposed approach supports more defensible allocation of ergonomic improvement resources and facilitates evidence-based justification of safety and production planning decisions.

The proposed framework makes a significant theoretical and practical contribution to the field of ergonomic risk management by integrating advanced fuzzy decision modeling with operational ergonomics. It furnishes manufacturing decision-makers with a structured, data-driven, and uncertainty-aware instrument capable of supporting sustainable production systems while enhancing worker health and safety.

Despite these contributions, several research directions remain to be explored. Firstly, subsequent studies should validate the proposed framework across multiple industrial contexts, including assembly lines, process industries, and service-oriented production systems, in order to assess its generalizability and sector-specific adaptability. Secondly, the integration of the framework with digital ergonomic monitoring systems, such as wearable sensors, vision-based posture analysis, and Industry 4.0-enabled data platforms, presents a promising avenue for transforming the model into a real-time or near-real-time decision-support tool. It is evident that extending the framework toward dynamic and longitudinal ergonomic assessment would facilitate decision-makers in tracking risk evolution over time and evaluating the long-term effectiveness of intervention strategies.

## Supplementary Information

Below is the link to the electronic supplementary material.

Supplementary Material 1.

## Data Availability

The author confirms that all the data generated or analyzed during this study are included in this published article (and its Supplementary Information files).

## Abbreviations

IVFFS: Interval-Valued Fermatean Fuzzy Sets
IVFF: Interval-Valued Fermatean Fuzzy
FFS: Fermatean Fuzzy Sets
IF: Intuitionistic fuzzy
PF: Pythagorean fuzzy
MCDM: Multi-criteria decision-making
PIPRECIA: Pivot Pairwise Relative Criteria Importance Assessment
MAIRCA: Multi-attributive ideal-real comparative analysis
EAM: Ergonomic assessment method
WMSDs: Work-related musculoskeletal disorders
MSDs: Musculoskeletal disorders
REBA: Rapid entire body assessment
RULA: Rapid upper limb assessment
OCRA: Occupational repetitive actions
QEC: Quick exposure check
SI: Strain index
OWAS: Ovako working posture analysis system

## Code availability

The study does not rely on custom-developed software or proprietary code. All computational procedures are based on established mathematical formulations of the IVFF–PIPRECIA, IVFF–MAIRCA, and Shannon ENTROPY methods, which are fully described within the manuscript. Therefore, the results can be reproduced using the provided equations and methodological steps. Any additional implementation details are available from the corresponding author upon reasonable request.
