# Supplementary material for: An uncertainty-aware ergonomic risk assessment framework using Interval-Valued Fermatean Fuzzy Sets and hybrid MCDM
Source: Sci Rep. 2026 Apr 4;16:16348. doi: 10.1038/s41598-026-46701-9 (PMC13213037; doi:10.1038/s41598-026-46701-9)
Supplement: Supplementary file 1 — Supplementary Material 1. [file 41598_2026_46701_MOESM1_ESM.docx]

**SUPPLEMENTARY INFORMATION**

**Table S1**. Overview of fuzzy and MCDM-based ergonomic risk assessment studies

| Author(s) | Year | Ergonomic Method(s) | Evaluation Focus | Sector | Solution Method(s)* | Decision Level | Main Contribution / Limitation |
| --- | --- | --- | --- | --- | --- | --- | --- |
| Delice and Can [14] | 2020 | Custom EAM | Lifting operations & human-related factors | Tube manufacturing | KEMIRA-M, BWM, MOORA, COPRAS | Risk factor weighting | Applies multi-criteria techniques to prioritize human-related risk factors; does not evaluate ergonomic method suitability. |
| Gumasing and Casela [39] | 2020 | REBA, NIOSH | Biomechanical risk assessment | Logistics | Ergonomic assessment methods | Single-method application | Uses established ergonomic tools for biomechanical assessment; lacks multi-layer decision integration. |
| Eyvazlou et al. [41] | 2021 | CMDQ, QEC, REBA | Demographic & physical risk factors | Dental profession | Ergonomic assessment methods | Risk factor evaluation | Examines demographic and physical risk factors; no fuzzy uncertainty modeling or method prioritization. |
| Koppiahraj et al. [12] | 2021 | EAWS, QEC, REBA, JSI | Workstation conditions & method selection | Industrial environments | Fuzzy VIKOR | Method comparison (single-layer) | Applies fuzzy VIKOR for workstation conditions; does not integrate uncertainty-aware layered architecture. |
| Wang et al. [30] | 2021 | REBA, RULA | Physical ergonomic risks | Industrialized construction | Fuzzy logic, 3D visualization | Risk visualization & detection | Integrates fuzzy logic with 3D visualization; no structured method selection framework. |
| Ozdemir et al. [16] | 2021 | DHM, ESM | Physical workload assessment | Refrigerator assembly line | MILP | Workload optimization | Uses MILP for workload balancing; does not address epistemic uncertainty in ergonomic evaluation. |
| Kose et al. [17] | 2022 | REBA, RULA, OWAS, OCRA, EAWS, QEC | **Multi-method comparative assessment** | Goods supplier industry | IVPF-AHP-TOPSIS | Multi-method comparison | Applies IVPF-AHP-TOPSIS; lacks interval-valued uncertainty modeling and layered decision structure. |
| Chatzis et al. [24] | 2022 | REBA | Postural risk classification | Industrial ergonomics | Deep learning | Posture classification / Real-time detection | Develops deep learning-based automatic risk classification; focuses on predictive ergonomics rather than structured method prioritization. |
| Junior et al. [15] | 2023 | OCRA | Repetitive task evaluation | Assembly line | MILP | Repetitive task evaluation | MILP-based workload modeling does not incorporate fuzzy epistemic uncertainty. |
| Hezam et al. [56] | 2023 | Multiple EAMs | Workstation prioritization | Industrial | IVFF–TOPSIS | Workplace/operation prioritization | Prioritizes ergonomic risk criteria using IVFF-based weighting; does not extend analysis to comparative method suitability or multi-layer decision integration |
| Kulaç and Kiraz [18] | 2024 | REBA, QEC, OCRA, COPSOQ | Physical, psychosocial & environmental factors | Wire harness factory | IVSF-AHP, MILP | Risk factor weighting | Uses IVSF-AHP; does not extend to method suitability evaluation |
| Sakinala et al. [27] | 2024 | Participatory ergonomics | Operator safety and ergonomic risk factors | Mining industry | Fuzzy modeling | Risk evaluation | Applies fuzzy modeling within participatory ergonomics for safety improvement; lacks structured comparison of ergonomic assessment methods. |
| Bernard and Varaprasad [42] | 2024 | REBA, NIOSH | Repetitive movement analysis | Drilling rigs | Simulation-based analysis | Simulation-based risk analysis | Simulation-based ergonomic evaluation; no multi-layer decision integration. |
| Tarakçi and Can [43] | 2024 | REBA, FMEA | Ergonomic risk prioritization of hazardous working | Production line | PF-VIKOR | Risk prioritization | Applies PF-VIKOR; no interval-based uncertainty modeling or method-level selection. |
| Mohd Nizan et al. [10] | 2024 | REBA, RULA | Human handling risk factors | Fabrication sector | Ergonomic assessment methods | Single-method application | Applies traditional ergonomic tools; lacks structured fuzzy integration. |
| Menanno et al. [23] | 2024 | 3D pose estimation | Real-time risk detection | HRC | AI-based monitoring | Real-time monitoring | Implements digital posture monitoring; lacks structured decision-support integration. |
| Mazloumi et al. [45] | 2025 | OWAS, RULA, REBA, customized technique | \| Comprehensive ergonomic risk assessment and method validation across multiple risk factor categories \| \| --- \| | Agriculture | Fuzzy Delphi, FAHP | Multi-method validation | Uses fuzzy Delphi and FAHP; no layered decision-support architecture. |
| Toktaş and Can [44] | 2025 | Custom ergonomic risk factors | Region-based ergonomic risk evaluation considering factor interactions | Dishwasher assembly line | FARE- KEMIRA-M | Region-based factor interaction modeling | Considers factor interactions; does not evaluate method suitability under uncertainty. |
| Tatar et al. [46] | 2025 | Fine–Kinney–based ergonomic risk assessment | Identification and prioritization of WMSD risks | Maritime port operations | Spherical fuzzy FUCOM–ARTASI | WMSD risk prioritization | Uses spherical fuzzy methods; does not address structured method selection |
| **Liu et al**. [55] | **2025** | REBA-based | Risk factor weighting | Manufacturing | IVFF–AHP | Risk factor weighting | Prioritizes ergonomic risk criteria using IVFF-based weighting; does not extend analysis to comparative method suitability or multi-layer decision integration |
| ***This study** | **---** | REBA, RULA, OCRA, QEC, SI, OWAS | Comparative ergonomic assessment under uncertainty | Machining | **IVFFS-PIPRECIA-IVFFS-MAIRCA-ENTROPY** | **Multi-layer method selection & operational prioritization** | **Introduces an integrated IVFF–PIPRECIA–MAIRCA–ENTROPY architecture for uncertainty-aware method prioritization and layered ergonomic decision support.** |
| ** Solution Method(s) refers to the decision-support or analytical framework applied, rather than the ergonomic assessment tools themselves.* | | | | | | | |

**Table S2.** Scale 1–2 for evaluation of criteria [53]

| Linguistic Terms | | IVFF Number | | | |
| --- | --- | --- | --- | --- | --- |
|  |  | $\boldsymbol{\mu}_{\boldsymbol{R}}^{\boldsymbol{L}}$ | $\boldsymbol{\mu}_{\boldsymbol{R}}^{\boldsymbol{u}}$ | $\boldsymbol{v}_{\boldsymbol{R}}^{\boldsymbol{L}}$ | $\boldsymbol{v}_{\boldsymbol{R}}^{\boldsymbol{u}}$ |
| Almost equal (E) | Scale 1-2 | 1,00 | 1,10 | 1,80 | 1,95 |
| Slightly more significant (SM) |  | 1,15 | 1,25 | 1,70 | 1,80 |
| Moderately more significant (MDM) |  | 1,30 | 1,40 | 1,44 | 1,65 |
| More significant (M) |  | 1,40 | 1,50 | 1,45 | 1,55 |
| Much more significant (MM) |  | 1,55 | 1,65 | 1,30 | 1,40 |
| Dominantly more significant (DM) |  | 1,70 | 1,80 | 1,15 | 1,25 |
| Absolutely more significant (AM) |  | 1,80 | 1,95 | 1,05 | 1,15 |
| Weakly less significant (WL) | Scale 0-1 | 0,80 | 0,95 | 0,05 | 0,15 |
| Moderately less significant (MDL) |  | 0,70 | 0,80 | 0,15 | 0,25 |
| Less significant (L) |  | 0,55 | 0,65 | 0,30 | 0,40 |
| Really less significant (RL) |  | 0,40 | 0,50 | 0,45 | 0,55 |
| Much less significant (ML) |  | 0,30 | 0,40 | 0,55 | 0,65 |
| Dominantly less significant (DL) |  | 0,15 | 0,25 | 0,70 | 0,80 |
| Absolutely less significant (AL) |  | 0,05 | 0,15 | 0,80 | 0,95 |

**Table S3.** Linguistic terms for assessing the ergonomic risk assessment methods (alternatives) [56].

| Linguistic Terms | IVFF Number | | | |
| --- | --- | --- | --- | --- |
|  | $\boldsymbol{\mu}_{\boldsymbol{R}}^{\boldsymbol{L}}$ | $\boldsymbol{\mu}_{\boldsymbol{R}}^{\boldsymbol{u}}$ | $\boldsymbol{v}_{\boldsymbol{R}}^{\boldsymbol{L}}$ | $\boldsymbol{v}_{\boldsymbol{R}}^{\boldsymbol{u}}$ |
| Very Very Low (VVL) | 0,05 | 0,10 | 0,90 | 0,95 |
| Very Low (VL) | 0,10 | 0,20 | 0,85 | 0,90 |
| Low (L) | 0,20 | 0,30 | 0,80 | 0,85 |
| Medium Low (ML) | 0,30 | 0,40 | 0,70 | 0,80 |
| Medium (M) | 0,40 | 0,50 | 0,60 | 0,70 |
| Medium-High (MH) | 0,50 | 0,65 | 0,50 | 0,60 |
| High (H) | 0,65 | 0,80 | 0,40 | 0,50 |
| Very High (VH) | 0,80 | 0,90 | 0,20 | 0,35 |
| Very Very High (VVH) | 0,90 | 0,95 | 0,05 | 0,10 |

**Table S4.** Ergonomic risk scores of operational steps obtained from multiple assessment methods

| Task  No | REBA Score | RULA Score | QEC  Score | OWAS Score | OCRA Score | SI  Score |
| --- | --- | --- | --- | --- | --- | --- |
| 1 | 6 | 5 | 69.14 | 2 | 10 | 10 |
| 2 | 4 | 4 | 70.37 | 3 | 9 | 9 |
| 3 | 5 | 4 | 72.22 | 3 | 8 | 10 |
| 4 | 5 | 6 | 70.37 | 3 | 12 | 10 |
| 5 | 4 | 4 | 71.6 | 3 | 10 | 9 |
| 6 | 6 | 5 | 72.22 | 2 | 8 | 9 |
| 7 | 4 | 4 | 70.37 | 2 | 9 | 9 |
| 8 | 7 | 6 | 65.43 | 4 | 11 | 10 |
| 9 | 5 | 5 | 70.37 | 3 | 10 | 9 |
| 10 | 5 | 6 | 77.16 | 3 | 13 | 11 |
| 11 | 6 | 5 | 65.43 | 4 | 12 | 10 |
| 12 | 4 | 4 | 77.16 | 3 | 9 | 9 |
| 13 | 7 | 6 | 76.54 | 4 | 12 | 11 |
| 14 | 6 | 5 | 70.37 | 3 | 10 | 10 |
| 15 | 6 | 5 | 70.37 | 3 | 10 | 9 |
| 16 | 6 | 5 | 71.6 | 4 | 12 | 10 |
| 17 | 4 | 4 | 70.37 | 3 | 9 | 9 |
| 18 | 5 | 5 | 72.22 | 3 | 10 | 10 |
| 19 | 5 | 6 | 70.37 | 3 | 12 | 10 |
| 20 | 4 | 5 | 76.54 | 3 | 11 | 10 |
| 21 | 5 | 5 | 70.37 | 3 | 10 | 9 |
| 22 | 6 | 6 | 74.69 | 3 | 12 | 11 |
| 23 | 5 | 5 | 65.43 | 2 | 9 | 9 |

**Table S5.** Operational steps in the production process

| Task No | Operational Step Name |
| --- | --- |
| 1 | Procurement of raw material for shafts (cast iron/steel) |
| 2 | Quality control of procured shafts |
| 3 | Cutting and shaping of shafts |
| 4 | CNC Machining of shafts (turning, milling, drilling) |
| 5 | Quality control of machined shafts |
| 6 | Procurement of raw material for forged holders |
| 7 | Quality control of procured raw material for holders |
| 8 | Forging process of holders |
| 9 | Quality control of forged holders |
| 10 | CNC Machining of forged holders |
| 11 | Formation of body draft (heating and pressing of shafts and holders) |
| 12 | Quality control of the body draft |
| 13 | Welding the center piece to the body draft |
| 14 | Welding quality control |
| 15 | Machining of holders on a universal lathe |
| 16 | Induction hardening of holders |
| 17 | Hardness control |
| 18 | Surface smoothing through grinding |
| 19 | Surface cleaning through sandblasting |
| 20 | Surface protection processes (painting) |
| 21 | Transfer to the assembly line |
| 22 | Assembly and final quality control |
| 23 | Packaging and preparation for shipment |
